# Theory of neuronal perturbome in cortical networks

**DOI:** 10.1073/pnas.2004568117

**Published:** 2020-10-14

**Authors:** Sadra Sadeh, Claudia Clopath

**Affiliations:** ^a^Bioengineering Department, Imperial College London, London SW7 2AZ, United Kingdom

**Keywords:** neuronal perturbation, cortical connectivity, sensory coding, visual cortex, perturbome

## Abstract

To unravel the functional properties of the brain, we need to untangle how neurons interact with each other and coordinate in large-scale recurrent networks. One way to address this question is to measure the functional influence of individual neurons on each other by perturbing them in vivo. Application of such single-neuron perturbations in mouse visual cortex has recently revealed feature-specific suppression between excitatory neurons, despite the presence of highly specific excitatory connectivity, which was deemed to underlie feature-specific amplification. Here, we studied which connectivity profiles are consistent with these seemingly contradictory observations, by modeling the effect of single-neuron perturbations in large-scale neuronal networks. Our numerical simulations and mathematical analysis revealed that, contrary to the prima facie assumption, neither inhibition dominance nor broad inhibition alone were sufficient to explain the experimental findings; instead, strong and functionally specific excitatory–inhibitory connectivity was necessary, consistent with recent findings in the primary visual cortex of rodents. Such networks had a higher capacity to encode and decode natural images, and this was accompanied by the emergence of response gain nonlinearities at the population level. Our study provides a general computational framework to investigate how single-neuron perturbations are linked to cortical connectivity and sensory coding and paves the road to map the perturbome of neuronal networks in future studies.

## Significance

Brains are composed of networks of neurons that are highly interconnected. A central question in neuroscience is how such neuronal networks operate in tandem to make a functioning brain. To understand this, we need to study how neurons interact with each other in action, such as when viewing a visual scene or performing a motor task. One way to approach this question is by perturbing the activity of functioning neurons and measuring the resulting influence on other neurons. By using computational models of neuronal networks, we studied how this influence in visual networks depends on connectivity. Our results help to interpret contradictory results from previous experimental studies and explain how different connectivity patterns can enhance information processing during natural vision.

Perturbative approaches to study neuronal dynamics are becoming pivotal in our understanding of the brain’s function and dysfunction (1–4). They however often involve perturbation of a large number of neurons, which renders the analysis of the underlying circuitry challenging. A more simplified approach that has been pursued recently is to map the functional influence of individual neurons by perturbing a single neuron at a time. Such single-neuron perturbations have recently revealed feature-specific suppression between excitatory neurons in mouse visual cortex (5). However, we still lack a mechanistic account of how these single-neuron functional influences are linked to cortical connectivity and dynamics, and how they can shed light on functional processing of realistic stimuli in large-scale cortical networks.

Specifically, how different motifs of excitatory (E) and inhibitory (I) connectivity interact with each other to give rise to functional properties of neuronal networks, and how this is manifested in single-neuron perturbations, remains unclear. For instance, several experimental studies have recently reported a highly specific pattern of connectivity in mouse primary visual cortex (V1), where excitatory neurons with similar functional properties (e.g., orientation selectivity) are connected together with higher probability and with stronger weights (6–9). This was suggested to give rise to feature-specific amplification of the feedforward input by the recurrent network (10, 11). The results of single-neuron perturbations, on the other hand, suggest that feature-specific suppression, rather than amplification, is the dominant mode of functional interaction between excitatory neurons (5). It has, therefore, remained puzzling how these seemingly paradoxical results should be interpreted and reconciled.

Here, we developed a theory of single-neuron perturbations and used computational modeling to shed light on these questions. We built and analyzed large-scale models of neuronal networks constrained with realistic receptive fields (RFs) and experimentally reported motifs of recurrent connectivity and studied the effect of single-neuron perturbations in these networks. Specifically, we asked which cortical connectivity regimes are consistent with the experimental results of single-neuron perturbations. Our results highlighted the crucial role of inhibitory connectivity patterns, and how they interact with excitatory motifs to give rise to feature-specific effects (e.g., amplification or/and suppression). We found that to obtain feature-specific suppression, strong and functionally specific subnetworks of E and I were necessary. That is, both E and I neurons with similar RFs should be connected together more strongly than their nonsimilar counterparts, which was consistent with recent results in visual cortex (12).

Our modeling results shed light on the above-mentioned controversy by showing that feature-specific amplification and suppression could both exist in the cortex, depending on the regime of functional similarity between the influencers and the influencees. Our model suggests specific predictions on how to observe this in the cortex. Computational modeling also helped us to formulate further predictions that experiments could not directly assess, for instance regarding the temporal evolution of influence and differential contributions of specific connectivity motifs. We could further link the result of single-neuron perturbations to sensory processing by studying how our model networks in different regimes encode and decode natural images. More generally, we show that our theory can be extended to study multiple-cell perturbations to map the perturbome of neuronal networks in future.

## Results

### Perturbations in Excitatory–Inhibitory Networks.

We first sought to analyze how single-neuron perturbations are linked to network connectivity and dynamics in simplified models. We asked how the perturbome of the network, that is the relative change in other nodes’ activity when a node is perturbed, is linked to the connectome, i.e., how strongly the nodes are connected together in their direct pathways. We expect a close match between the two for primarily feedforward structures or networks with weak recurrent coupling. However, in neuronal networks with strong recurrent coupling the complex interaction of excitation and inhibition and the emergent dynamics of the network may change the perturbome.

**Fig. 1.**
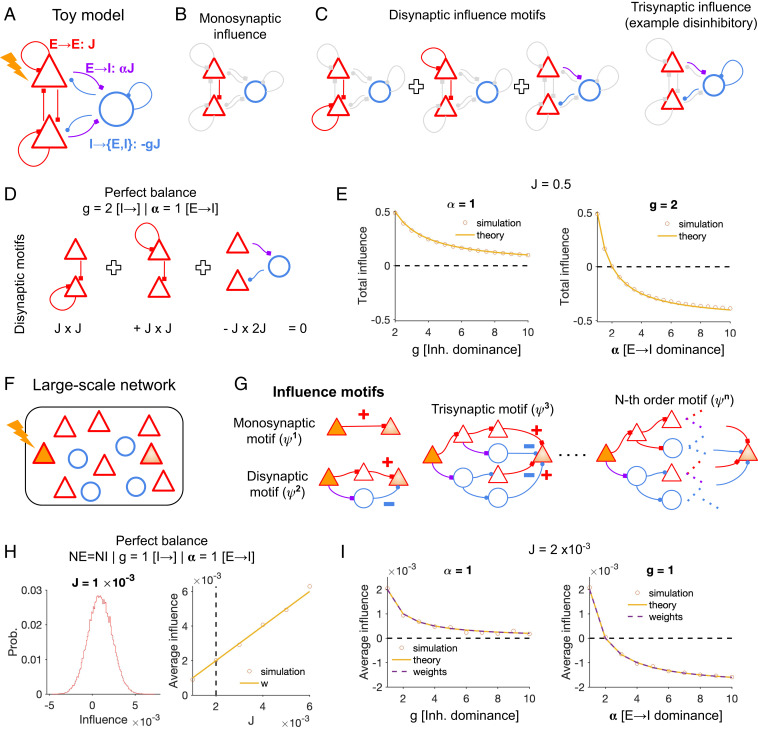
Influence of perturbations in excitatory–inhibitory networks. (*A*) A reduced circuit model composed of two excitatory (E, red) and one inhibitory (I, blue) subpopulations. One E subpopulation (E1, *Top*) is perturbed and the influence of this perturbation on the activity of the other E subpopulation (E2, *Bottom*) is studied. (*B*) The direct effect of perturbation is highlighted via the monosynaptic connection from E1 to E2. (*C*) The effects of disynaptic interactions from E1 to E2 are highlighted with three possible motifs (E1→E2→E2, E1→E1→E2, and E1→I→E2). An example trisynaptic motif is shown on the *Right*, which has a disinhibitory effect overall. (*D*) The contributions of disynaptic motifs to the influence cancel each other out under a perfect balance of excitation and inhibition. This is obtained by α = 1 and g=2, with the latter compensating for the smaller size of inhibitory neurons compared to two E subpopulations. (*E*) Total influence of E1 perturbations on E2 activity in rate-base simulations (circles) for different values of g. Keeping α=1 and increasing g leads to divisive inhibition of the initial influence, but the influence remains positive (*Left*). Increasing α, on the other hand, can lead to suppressive influence for higher values (*Right*). The prediction from the theory is plotted with solid lines in each case (*J* = 0.5). (*F*) Influence is evaluated in large-scale random networks of excitatory and inhibitory neurons. (*G*) The effect of perturbation of an E neuron (the influencer) on another E neuron (the influencee) in the network can be mediated by multiple pathways, including monosynaptic and higher-order motifs. The sign of the net influence at each branch is determined by considering the interaction of the signs of all synapses in the respective pathway. (*H*, *Left*) Distribution of influence between all pairs of excitatory neurons in the network, in the case of perfect balance (α=1 and *g* = 1, NE = NI = 500, and *J* = 0.001). (*H*, *Right*) Average influence in the networks with different values of *J*. Given perfect balance, the average influence always matches with the average direct connection weight between E neurons, even for networks with unstable excitatory subnetworks (the border of instability is shown by the dashed line). (*I*) Average influence in networks with *J* = 0.002 and different values of *g* and α. Similar behavior to E is observed, where increasing *g* leads to divisive inhibition and increasing α is needed to obtain suppressive influence. The prediction from the weight matrix of the network, and from the theory (*SI Appendix*, *Methods* and Eq. 80), match with the results of rate-based simulations.

We started our study by analyzing a simplified network composed of two excitatory (E) and one inhibitory (I) subpopulations (Fig. 1*A*). In weakly coupled networks, the influence of perturbing an E subpopulation (E1; influencer) on the other (E2; influencee), is mainly determined by the direct, monosynaptic connection between the two (E1→E2; parameterized by J) (Fig. 1*B*). For strongly coupled networks, however, the effect of higher-order interactions become more prominent. Three disynaptic influence motifs possible for this network are shown in Fig. 1*C*, with two of them (E1→E1→E2 and E1→E2→E2) conferring a net excitatory influence and the other (E1→I→E2) exerting a net inhibitory effect. Influence motifs of higher orders can contain more complicated interactions, including disinhibitory motifs (as highlighted by an example trisynaptic motif E1→I→I→E2 in Fig. 1*C*). The total influence in the network, therefore, depends on the exact summation of all these excitatory and inhibitory pathways.

For linear networks (e.g., linearized rate-based or spiking networks), the influence of perturbing E1 on E2, ψ(E1→E2), can be analytically obtained from the weight matrix W by the operator $A={\left(1-W\right)}^{-1}$ (*SI Appendix*, *Methods* and Eq. 15). For the network with connection weights as parameterized in Fig. 1*A* it can be computed as follows:

$$\psi \left(E1\to E2\right)=\frac{J+gJ^{2}\left(1-\alpha \right)}{1+J\left(g-2\right)+2J^{2}g\left(\alpha -1\right)}.$$

Here, g (inhibition dominance) and α (E→I dominance) denote, respectively, the strength of inhibitory synapses and E→I weights relative to E→E connections (J). “Perfect balance,” given by α=1 and *g* = 2 (since there is only one I subpopulation), implies that ψ(E1→E2)=J. In this case, we can say that the effective influence of perturbations in the network (or the perturbome) is similar to the weight of direct connections (J) that can be inferred from the network connectome. An intuitive explanation is that all higher-order motifs cancel each other in terms of their positive and negative effects. This is shown for disynaptic motifs in Fig. 1*D* and can be worked out for higher-order motifs in the same fashion.

For most realistic conditions, however, the assumption of perfect balance might not hold [e.g., neuronal networks in mouse V1 with dense and strong E–I interactions (13–15)]. We therefore need to analyze how the influence changes when we vary the key parameters of E–I connectivity. To study this, we kept either α (E→I dominance) or g (inhibition dominance) the same as before and changed the other parameter (Fig. 1*E*). Our simulations showed that increasing g alone decreased the influence in a divisive manner: the stronger the g, the weaker the influence between E1 and E2, but it remained always positive (Fig. 1*E*). This was consistent with the analytical expression above, when we let α=1, leading to $\psi \left(E1\to E2\right)=J/\left(1+J\left(g-2\right)\right)$. When we fix g and change α instead, negative influence can be obtained for large values of α (Fig. 1*E*). This was also obtained from our analytical expression under the specific condition of g=2, leading to $\psi \left(E1\to E2\right)=\left(J+2J^{2}\left(1-\alpha \right)\right)/\left(1+4J^{2}\left(\alpha -1\right)\right)$. In contrast to the previous case, now the numerator can take negative values, provided α>1+1/(2J). Our analysis thus shows that, while strong inhibition dominance (g) can scale the influence between two E subpopulations and enable a divisive inhibition, strong g and α (E→I dominance) can change the sign of E→E influence and lead to suppressive effects.

We next asked whether our basic insights obtained from this simplified circuit can be generalized to large-scale recurrent networks composed of many E and I neurons (Fig. 1*F*). We simulated the effect of perturbing a single E neuron in rate-based networks with random weights and measured the influence of single-neuron perturbations on other E neurons. The influence ψ(E1→E2) can be exerted via the monosynaptic connection, but also through many indirect, higher-order influence pathways with a net excitatory or inhibitory effect (Fig. 1*G*). To obtain the total influence in the network, we therefore need to account for all these pathways. For a weight matrix with similar parameterization of average E–I connectivity as Fig. 1*A*, we calculated the net influence between two E neurons (*SI Appendix*, *Methods* and Eq. 88):

$$\psi \left(E_{i}\to E_{j}\right)=\frac{J+g\text{N}J^{2}\left(1-\alpha \right)}{1+\left(g-1\right)NJ+g\left(\alpha -1\right)N^{2}J^{2}}.$$

We first tested the condition of perfect balance (α=1, g=1), as discussed above. Although we obtained a range of influences between different pairs of neurons due to randomness of weights, the average influence in network simulations was similar to the weight of direct connectivity, *J* (Fig. 1*H*). This was also the case for large values of *J*, which would render the E subnetwork unstable in the absence of inhibition, meaning that, although higher-order E motifs can be unstable and nonconverging, they are matched by a similar inhibitory feedback, which cancels them at all higher orders. This result was consistent with our analytical finding, whereby allowing for α=1 and g=1 cancels all additional terms and leads to $\psi \left({\text{E}}_{i}\to {\text{E}}_{j}\right)=J$. Deviations from perfect balance, by fixing α=1 or g=1 and changing the other parameter, led to similar qualitative results as before (Fig. 1*I*): increasing inhibition dominance (*g*) alone led to divisive effects, while large values of E→I dominance (*α*) were needed for changing the sign of influence. Both effects were well captured by the weight matrix [when calculating the influence numerically from $\psi ={\left(1-W\right)}^{-1}$] and by our analytical expression (Fig. 1*I*). We therefore conclude that the regimes of influence, and their dependence on different components of connectivity, can be described by our theoretical framework in both small and large-scale E–I networks.

### Single-Neuron Perturbations in Large-Scale Networks of Visual Cortex.

**Fig. 2.**
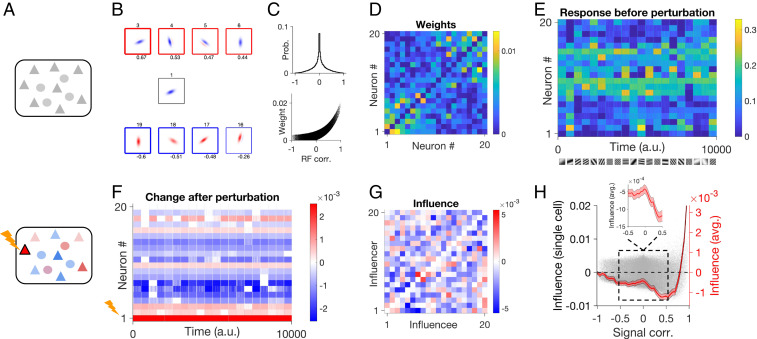
Influence of single-neuron perturbations in large-scale neuronal networks. (*A*) Large-scale networks composed of excitatory (triangles) or inhibitory (circles) neurons are simulated in the baseline state (above) or after perturbing a single neuron (*Lower*). (*B*) Example visual receptive fields (RFs) of excitatory neurons. Sample neurons with positive (*Upper*, red squares) and negative (*Lower*, blue squares) RF correlations (CC) with the RF in the *Center*. The thickness of lines around each RF is proportional to the absolute value of RF CC (indicated on the *Bottom*). IDs of example neurons (indicated on *Top* of each RF) are kept the same in the rest of the figure, for comparison. (*C*) Distribution of RF correlations for all excitatory pairs in the network (*Upper*), and the relationship between connection weights and RF correlations for the respective pairs (*Lower*). (*D*) Sample weight matrix for 20 example excitatory neurons. Neurons are sorted according to their similarity (RF CC) to RF #1. (*E*) Firing rate response of example neurons to example static gratings (shown on the *Bottom*). (*F*) Change in the response of neurons after perturbing neuron #1. (*G*) Influence (average response change normalized by the perturbation size) for all pairs of example excitatory neurons as influencers (different rows) or influencees (different columns). (*H*) Influence as a function of signal correlation for all excitatory pairs (gray dots). The average influence at different levels of signal correlation is plotted on the *Right* (bin size: 0.05). Shading denotes ± SEM. (*Inset*) Zoom into the intermediate range of RF similarity. $N_{\text{E}}=N_{\text{I}}=400,\;J_{\text{EE}}=0.0025,\;J_{\text{EI}}=0.005\left(\text{α}=2\right),\;J_{\text{IE}}=J_{\text{II}}=-0.005\left(g=2\right),\;\eta_{\text{EE}}=\eta_{\text{EI}}=\eta_{\text{IE}}=\eta_{\text{II}}=2,\;\tau =10$.

To go beyond random networks and relate our analysis to more biologically realistic networks, we next studied the effect of single-neuron perturbations in large-scale network models of visual cortex (Fig. 2*A*). Individual excitatory and inhibitory neurons were modeled by two-dimensional visual RFs with randomly assigned initial parameters (e.g., preferred orientations and spatial frequencies) (Fig. 2*B*). In accordance with experimental findings (8), the connectivity of neurons in the network was governed by a RF similarity-based rule, where neurons with more similar RFs had stronger connection weights (Fig. 2 *C* and *D*).

We first simulated the responses of the network in the baseline state (i.e., before perturbation) (Fig. 2 *A*, *Upper*), in response to gratings of different orientations and spatial frequencies (Fig. 2*E*). Neurons showed a wide range of responses to stimuli, with skewed responses for excitatory neurons and larger responses for inhibitory neurons, on average (*SI Appendix*, Fig. S1*A*). Excitatory neurons responded with various degrees of selectively to specific features of stimuli, like their orientation (*SI Appendix*, Fig. S1*B*). Similarity of neuronal responses can be assayed by the correlation of their responses to stimuli (response or signal correlations; see *SI Appendix*, *Methods*). We observed a wide range of signal correlations between E and I pairs, and this signal correlation was generally expected from respective RF correlations (*SI Appendix*, Fig. S1 *C* and *D*).

To obtain single-neuron influences, we then simulated the response of the network with extra perturbations of a single excitatory neuron (“influencer”) (Fig. 2 *A*, *Lower*) and measured the change in the activity of other excitatory neurons (“influencees”) (Fig. 2*F*). The average response change of each influencee as a result of perturbation normalized by the strength of perturbation was taken as a measure of the functional “influence” (Fig. 2*G* and *SI Appendix*, Fig. S1*E*). To investigate how the interaction between neurons depends on their similarity, we plotted the influence for each pair of neurons (influencers and influencees) against their signal correlation (Fig. 2*H*). For moderate correlations, the net influence was negative, consistent with the average negative effect of single-neuron perturbations in experiments (5). Moreover, we observed “feature-specific suppression” in this regime, that is the negative influence was stronger for pairs with more similar response properties, on average (Fig. 2 *H*, *Inset*). These results are consistent with feature-specific suppression observed in single-neuron perturbations in vivo (5).

However, this behavior changed for pairs with very strong response correlations, where we observed a positive influence, on average (Fig. 2*H*). A similar trend had been observed for high “trace correlations” in the experiments (cf. figure 5B in ref. 5). Based on our results, this regime of amplification is linked to RF similarity of neuronal pairs and hence can be assumed as “feature-specific amplification.” At the population level, these positive influences were stronger but less frequent, while the main bulk of influence between neuronal pairs was negative and small (*SI Appendix*, Fig. S1*E*). These results therefore suggest different regimes of influence in the networks, whereby pairs of neurons with moderate response similarity (most pairs) show feature-specific suppression on average, while feature-specific amplification is dominant for highly similar RFs (rare examples).

### Cortical Connectivity and Single-Neuron Influence.

**Fig. 3.**
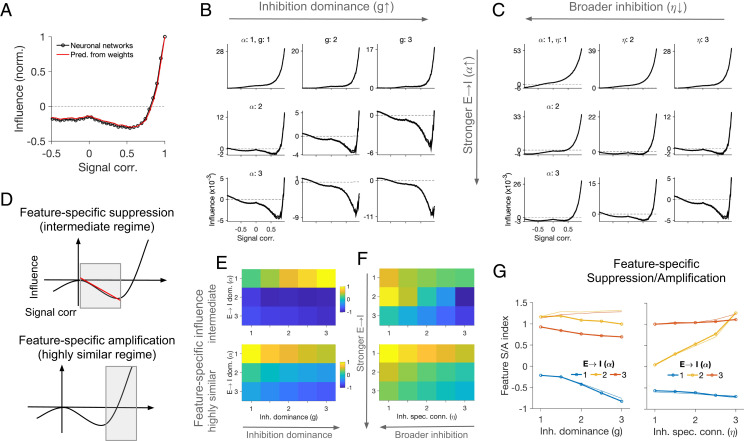
Connectivity regimes and feature-specific influence. (*A*) Average influence as a function of signal correlation in neuronal networks (as reported in Fig. 2*H*) and the theoretical prediction of this influence from the weight matrix of the network. (*B*) The average influence as a function of signal correlations inferred from the weight matrices of networks with different parameters of E→I connectivity (*α*) and inhibition dominance (*g*). (*C*) Same as *B* for different strength of E→I connectivity (*α*) and specificity of I→{E,I} connections (*η*). (*D*) Each pattern of influence was quantified by analyzing the degree of feature-specific amplification in the intermediate regime, quantified by the slope of the linear fit to the curve (*Upper*) and the average amount of feature-specific amplification in the highly similar regime (*Lower*). (*E*) Feature-specific influence for intermediate (*Upper*; as described in Fig. 3*D*, *Upper*) and highly similar regime (*Lower*; as described in Fig. 3*D*, *Lower*) of neuronal similarity for different combination of inhibition dominance (*g*) and strength of E→I connectivity (*α*) in the network. (*F*) Same as *E* for different strength of E→I connectivity (*α*) and specificity (*η*) of I→{E,I} connections. (*G*) Feature-specific suppression/amplification (S/A) index, combining both aspects of suppression and amplification at different regimes, for different combination of parameters. The thicker lines show the values inferred from the weight matrix, and the thinner lines correspond to simulations of the neuronal networks (*SI Appendix*, Fig. S2).

To better understand how these feature-specific effects emerge, and how they are related to cortical connectivity, we used our theoretical framework (as described above) for the analysis of single-neuron perturbations (Fig. 3). For linear networks, the theory can predict the impact of single-neuron perturbations on other neurons as a function of the weight matrix, so we can evaluate the average influence of neuronal pairs in the same networks as a function of their similarity. The numerical prediction from the weight matrix shows the same nonmonotonic behavior as our previous simulations in rate-based networks (Fig. 2*H*), with feature-specific suppression for moderate response correlations and feature-specific amplification for highly similar RFs (Fig. 3*A*). We therefore conclude that the main properties of feature-specific suppression/amplification arising from single-neuron perturbations can be inferred from the weight matrix, when considering the effect of monosynaptic and higher-order pathways of E–I interactions (Fig. 1*G*).

To see how the key parameters of connectivity change this behavior, we varied inhibition dominance (g) and E→I dominance (α) in the network and characterized the influence in each case. Consistent with our analysis before (Fig. 1), increasing inhibition dominance alone did not introduce any feature-specific suppression; however, when this was combined with strong E→I connections, feature-specific suppression emerged (Fig. 3*B*). Feature-specific amplification for highly similar RFs was present for moderate values of *g* and ⍺, but became less prominent for large values (Fig. 3*B*). Our theoretical analysis also suggested that broad inhibition alone does not confer feature-specific suppression (*SI Appendix*, *Methods*), a finding that was further confirmed in simulations with broader inhibitory connectivity (parameterized by η, controlling the specificity of inhibitory connections; see *Methods*) (Fig. 3*C*). Simulation of neuronal networks with similar connection weights led to similar results, for both inhibition dominance and broad inhibition scenarios (*SI Appendix*, Fig. S2).

To systematically characterize the functional properties of our networks, we developed a metric (Feature S/A index; *SI Appendix*, *Methods*) that quantifies the simultaneous presence of feature-specific suppression at intermediate regimes and feature-specific amplification for highly similar regimes (Fig. 3*D*). Higher values of this index correspond to stronger feature-specific suppression at moderate levels of RF similarity and feature-specific amplification for highly similar RFs (Fig. 3 *E* and *F*). Consistent with our qualitative observation before (Fig. 3 *B* and *C*), neither inhibition dominance nor broad connectivity of inhibition resulted in high values of this index, in the absence of strong E→I connectivity (Fig. 3*G*). Quantifying the functional behavior of rate-based networks with different parameters (*SI Appendix*, Fig. S2) led to very similar results (Fig. 3*G*). Consistent with our analysis before, these results therefore suggest that strong and specific inhibition and E→I connectivity are necessary and sufficient conditions to obtain patterns of functional influence similar to the experimental results.

### Contribution of Different Motifs to the Influence.

**Fig. 4.**
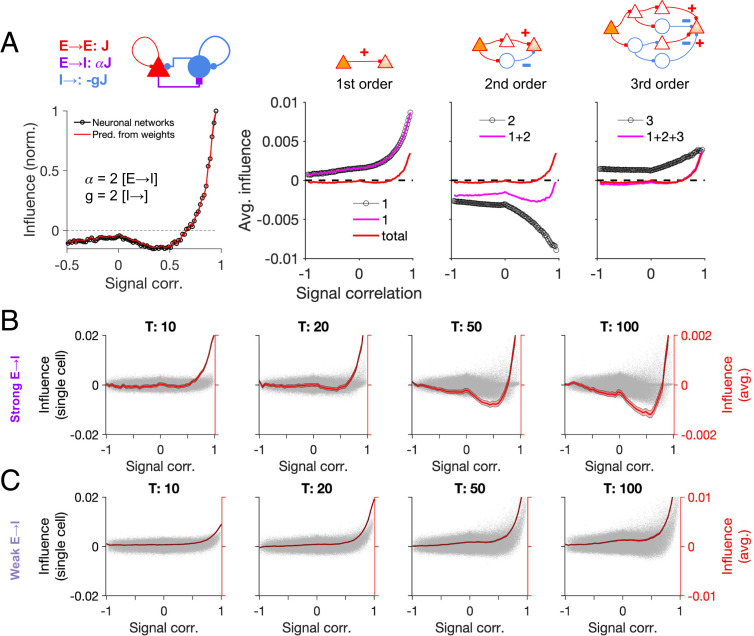
Contribution of different network motifs to influence in excitatory–inhibitory networks. (*A*, *Left*) The average total influence as a function of signal correlation, normalized to the maximum influence. (*A*, *Right*) Average influence is calculated for different motifs of the connection weights (inferred from the corresponding power of the weight matrix, W), up to the third order. Circles in each plot show the average influence of the respective motif, with magenta showing the sum of motifs up to that order. The total influence (red; same as the red curve on *Left*) is overlaid for comparison. $J_{\mathrm{EE}}=0.0014,\;J_{\mathrm{EI}}=0.0028\left(\text{α}=2\right),\;J_{\mathrm{IE}}=J_{\mathrm{II}}=-0.0028\left(g=2\right)$; other parameters are the same as in Fig. 2. (*B*) Influence as a function of signal correlation in neuronal networks (similar to Fig. 2*H*), when the influence is inferred from the average firing rate up to time *T* (denoted on *Top* of each plot). Gray dots: all pairs; red: average influence (bin size, 0.05). Shading denotes ± SEM at each bin. α=2, g=2, η=3; other parameters the same as Fig. 2. (*C*) Same as *B* for a network with weaker E→I connectivity (α=1, g=2, η=3).

Our analysis so far highlighted the key role of connectivity in feature-specific interactions. To further shed light on how different components of connectivity give rise to suppressive or amplifying effects, we analyzed the contribution of different influence motifs. To this end, we calculated how the average influence would depend on the signal correlation if it were limited to certain motifs (Fig. 4*A*). The *n*th-order influence was calculated from the *n*th power of the weight matrix $\left(W^{n}\right)$ (*SI Appendix*, *Methods* and Eq. 21).

As expected, first-order interactions only conveyed feature-specific amplification. Feature-specific suppression emerged in the second-order influence motifs, but this pattern was reversed in the third-order motif, which again showed feature-specific amplification (Fig. 4*A*). The net effect at intermediate regimes, however, remained negative, due to stronger feature-specific suppression of the second-order influences. Similar analysis can be repeated for motif-specific influences in networks with different combinations of α and g (*SI Appendix*, Fig. S3), revealing how feature-specific suppression can emerge in certain regimes (Fig. 3 *B* and *C*). Specifically, it shows that the sharpness of feature-specific suppression depends on the strength and specificity of E→I connections (Fig. 4*A* and *SI Appendix*, Fig. S3), which is evident for instance from the E→I→E pathway of the second-order motif (Fig. 4*A*). As feature-specific suppression is emerging as a higher-order motif, we would expect it to be broader than monosynaptic amplification. That is, even if all of the properties of E and I neurons were matched (same RFs and connectivity profiles), broadening of inhibitory influence could still arise from averaging over intermediate nodes.

The contribution of different motifs can also be observed in the temporal evolution of the total influence. In our previous analyses, we discarded the transient activity and evaluated the influence from neuronal responses in the stationary state. However, transient responses can reveal important insights about the operation of neuronal networks, especially how neuronal interactions evolve over time to shape the influence. We therefore analyzed the influence as inferred from the average activity at different time intervals after single-cell perturbations (Fig. 4*B*). Feature-specific amplification for very high response correlations was evident from very early responses, arguing for its monosynaptic nature (Fig. 4*A*). Feature-specific suppression, on the other hand, emerged and strengthened over time, consistent with its polysynaptic nature (Fig. 4 *A* and *B*). Notably, such dynamics could not be observed in networks with weak E→I connectivity (Fig. 4*C*). These results thus shed light on the evolution of the influence over time and are consistent with the contribution of higher-order motifs to feature-specific suppression.

### Influence as a Function of Individual Features of Receptive Fields.

**Fig. 5.**
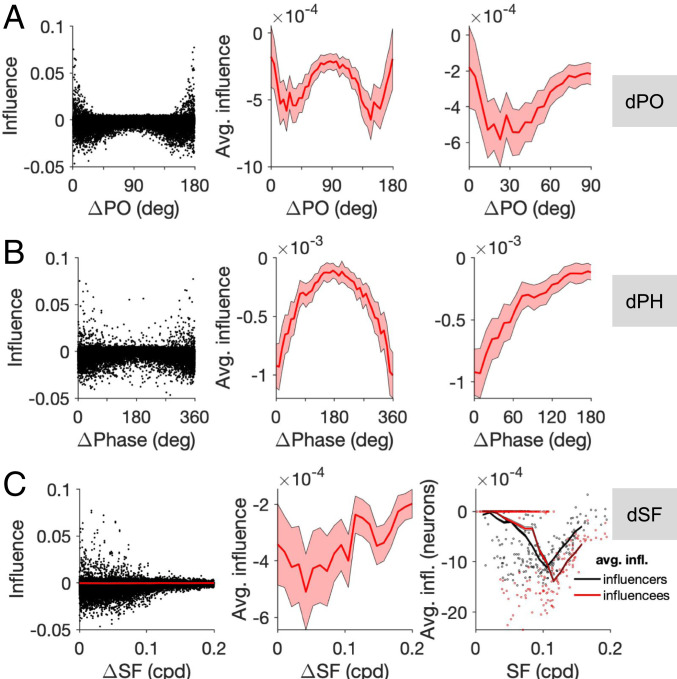
Influence as a function of individual features. (*A*) Influence (inferred from perturbing neuronal networks similar to Fig. 2) as a function of the difference in the preferred orientation (dPO) of neuronal pairs. (*Left*) Distribution of all pairs. (*Middle*) Average influence as a function of dPO (in bins of 4.5°). (*Right*) Zoom in (*x* axis) of the average influence to highlight the nonmonotonic pattern. (*B*) Same as *A* for the difference in preferred spatial phase (dPhase). Bin size, 9°. (*C*) Influence as a function of the difference in the preferred spatial frequency (dSF) of neuronal pairs. (*Left*) Distribution of all pairs (black) and the average influence (red). (*Middle*) Average influence as a function of dSF (in bins of 4.5°). (*Right*) Average influence per neuron [resulting from perturbing the neuron as an influencer (black) or observed by the neuron as an influencee (red)], as a function of its preferred SF. Dots show the result for all neurons in the network, and lines denote the average at each SF. Bin width, 0.01.

We presented the results of single-neuron perturbations in terms of similarity of neuronal responses, as we had access to actual RFs of neurons in our model networks. However, mapping the full RF of neurons in experiments is not always feasible, and experimental results are often expressed in terms of marginal feature selectivity of neurons (e.g., their tuning to individual features of RFs like preferred orientation or spatial frequency). To relate better our results to such experiments (e.g., as in ref. 5), we analyzed the influence as a function of individual features of neurons (Fig. 5).

#### Preferred orientation.

Characterization of the influence as a function of the difference between the preferred orientation (PO) of the influencers and influencees revealed feature-specific suppression for intermediate PO differences (dPOs), where more suppression was observed between pairs with more similar POs (Fig. 5*A*), in keeping with experimental results (cf. figure 3*I* in ref. 5). However, our analysis revealed an opposite trend of feature-specific amplification (i.e., less suppression for pairs with more similar POs) for very small differences (Fig. 5*A*). That is a consequence of feature-specific amplification for the regime of highly similar RFs as we described before (Figs. 2*H* and 3), when that similarity is projected over an individual feature of RFs, namely their PO. Our results thus suggest that mapping the dPO of neuronal pairs with more resolution and/or larger sample size should reveal another regime of amplification, in addition to feature-specific suppression for the intermediate range.

#### Spatial phase.

Among the individual features of the RFs, we found that the preferred spatial phase of neuronal pairs revealed the strongest feature-specific suppression. Plotting the influence as a function of the difference in preferred spatial phase (dPH) of neuronal pairs revealed a monotonic increase with dPH, indicating that neuronal pairs with the closest preferred spatial phase show the most feature-specific suppression, on average (Fig. 5*B*). As this aspect has not been explored in the experiments (5), our results suggest that analysis of influence as a function of phase difference could reveal a very strong dependence of influence on this feature.

#### Spatial frequency.

We also analyzed the dependence of influence on the difference in the preferred spatial frequency (dSF) of neuronal pairs (Fig. 5*C*). Here, the relationship was less obvious and noisier, especially for small to moderate dSFs (<0.1, Fig. 5*C*). This is consistent with the experimental results, which did not reveal a significant dependence of feature-specific suppression on dSF. However, we found a significant bandpass dependence of influence, when we calculated the average influence for each neuron, either as an influencer (i.e., the average influence resulting from the neuron to all influencees) or as an influencee (i.e., the average influence experienced by the neuron from all influencers), as a function of the neuron’s preferred SF (Fig. 5*C*).

#### Interaction of individual features.

We next analyzed how the interaction of above-mentioned features is related to the influence. That is, instead of analyzing the influence as a function of a single feature, we studied its changes in the space of multiple features (*SI Appendix*, Fig. S4). We analyzed the influence as a function of the conjoint distribution of differences in PO and phase (*SI Appendix*, Fig. S4*A*) or spatial frequency (*SI Appendix*, Fig. S4*B*). The bandpass dependence of influence on dPO that we observed before (*SI Appendix*, Fig. S4*A*) was exacerbated for small differences in both cases (small dPH and dSF regimes), and vanished for the regimes with large differences (*SI Appendix*, Fig. S4 *A* and *B*). This suggests that controlling for the difference in other properties of the cells (individual features of their RFs here, e.g., SF or spatial phase), can amplify the effect of feature-specific influence for unique individual features. Analysis of the dependence of the influence on dPO, when controlling for dSF and dPH at the same time, revealed similar results (*SI Appendix*, Fig. S4*C*).

### Pattern of Influence in Networks with Different Levels of Realism.

We next asked whether our results hold in networks with different levels of biological realism. We simulated and analyzed spiking networks, networks with sparse connectivity, and networks with more realistic inhibition.

#### Spiking networks.

Spiking neurons are more nonlinear than rate-based units, and the response of spiking networks can depend on the operating regime of the network (16, 17). Especially, the activity of spiking networks is more noisy in the fluctuation-driven regime of activity with a strong balance of excitation and inhibition. We therefore asked whether our results hold in balanced spiking networks (*SI Appendix*, Fig. S5). The network activity was much more noisy than rate-based simulations, and we therefore needed significantly longer simulations to obtain reliable estimates of the influence. However, it was possible to observe our key findings in these networks (*SI Appendix*, Fig. S5*F*). We also inferred the influence from the membrane potential (Vm) of neurons and found that feature-specific suppression was more evident in Vm-based influences (*SI Appendix*, Fig. S5*F*). Our results thus suggest that pattern of feature-specific influence can be observed from both spiking activity and Vm, and that future perturbation studies can employ voltage recordings to better study influence in sparsely active networks.

#### Networks with sparse connectivity.

We assumed all-to-all connectivity in our simulations so far and only changed the weight of connections, either randomly (Fig. 1 *F*–*I*) or according to neuronal selectivity (Fig. 2). While local inhibitory connectivity (I→E as well as E→I) has been reported to be very dense and approaching all-to-all connectivity (14, 18, 19), E–E connectivity in local networks is sparse. We therefore asked whether this sparse connectivity changes our results (*SI Appendix*, Fig. S6). Overall, we observed similar results in the average behavior of the network (*SI Appendix*, Fig. S6*A*). However, a bimodal distribution of influence emerged, when we investigated the behavior of individual pairs (*SI Appendix*, Fig. S6*B*). This result thus predicts an increase in the variance of influence for higher signal correlations, a pattern that seems to exist in the experimental data too (cf. figure 5B in ref. 5). Further analysis revealed that this dichotomy arises from connectivity: Direct connections showed primarily amplification-dominated behavior, while suppressive effects mainly emerged in unconnected pairs (*SI Appendix*, Fig. S6 *C* and *D*). This again emphasizes the polysynaptic nature of feature-specific suppression (Fig. 4). Our results therefore suggest that the connectivity between E–E pairs should be related to their feature-specific influence in a specific way. If experiments were biased toward the connected pairs, they would observe more amplification effects and may underestimate the significance of suppressive influences.

#### Networks with more realistic inhibition.

We also tested our results in networks with more realistic architecture of inhibition. We specifically tested 1) networks with biological fractions of E and I neurons; 2) networks with different time course of inhibition; and 3) networks with broader tuning of inhibition, and found that our basic results hold in all variants (*SI Appendix*, Fig. S7).

1) We first reduced the number of inhibitory neurons to biological ratio (E: 80%; I: 20%). This initially changed the behavior of the average influence as a function of response similarity (*SI Appendix*, Fig. S7 *A* and *B*), but when the weights of inhibitory neurons (I-to-E and I-to-I) were increased to compensate for the reduction in their size (13, 18), the original pattern was recovered (*SI Appendix*, Fig. S7*B*). These results demonstrate that as long as there is a minimum number of inhibitory neurons in the circuit to have enough diversity of RFs, and the strength of inhibitory feedback is strong enough, inhibition is capable of providing feature-specific feedback, which leads to feature-specific suppression. However, in the absence of such strong feedback, we should expect a shift toward amplification in feature-specific influence.
2) Next, we changed the time constant of inhibition (twice faster or slower as excitation) and observed similar results (*SI Appendix*, Fig. S7*C*). This might be expected as our results already had a good match with the theoretical prediction from the weight matrix, which does not account for any temporal dynamics by definition.
3) Finally, we studied networks with broader inhibition (*SI Appendix*, Fig. S7*D*). We implemented this in two ways: 3a) by allowing for broader tuning of inhibition, or 3b) by making the inhibitory weights less specific (broader connectivity).
3a) We generated inhibitory neurons with a range of RFs, which were on average less elongated (and hence less orientated) than excitatory RFs (*SI Appendix*, Fig. S7 *E* and *F*). Our simulations showed that similar results can be obtained with the new configuration (*SI Appendix*, Fig. S7*D*), arguing that a range of inhibitory selectivity with broader properties might be tolerated. This is in fact consistent with a broad range of orientation selectivity reported in inhibitory neurons (see, e.g., refs. 20 and 21).
3b) We also changed the specificity of connection weights for inhibitory connections (I-to-I, I-to-E, and E-to-I) to obtain broader feature-specific connectivity. We found that the result was more susceptible to this parameter (*SI Appendix*, Fig. S7*D*). Based on this result, we can conclude that although inhibitory neurons can have broader RFs, their weights as a function of their response/RF similarity should still be sharp (especially E→I; not shown).

### Inhibitory Single-Neuron Influence.

We also studied the influence when inhibitory neurons are perturbed as influencers instead of excitatory neurons. Mapping such inhibitory influences is more challenging experimentally, but we could investigate it in our model (*SI Appendix*, Fig. S8). We observed stronger negative influences, on average (*SI Appendix*, Fig. S8), presumably due to stronger weights of I→E connections. Beyond the mean suppression, a negative slope of influence versus response correlation (which indicates feature-specific suppression) was observed for higher signal correlations (*SI Appendix*, Fig. S8*A*), as opposed to excitatory single-cell perturbations where such a feature-specific suppression was present in the intermediate regime (illustrated in Fig. 3*D*). In fact, lack of a significant negative slope for intermediate positive signal correlations indicated an absence of feature-specific suppression in this regime (*SI Appendix*, Fig. S8*B*). The negative slope was, however, present for negative signal correlations in this regime, leading to some degree of apparent feature-specific “amplification” for intermediate negative correlations (*SI Appendix*, Fig. S8*B*). Thus, the pattern of inhibitory influence in the intermediate regime seems to be the opposite of the pattern of excitatory influence (cf. Fig. 2*H*), while the feature-specific amplification for highly similar regime is obviously missing. Another conspicuous difference between excitatory and inhibitory single-neuron perturbations was in the transient responses of the latter, which did not show a significant change in overall shape over time (*SI Appendix*, Fig. S8*C*). This argues that polysynaptic motifs are more important in establishing feature-specific suppression in excitatory single-neuron perturbations, as it involves the interaction of excitation and inhibition.

### Influence Resulting from Multiple-Neuron Perturbations.

Our results in the previous section revealed how interaction of multiple features can shed light on additional properties of functional influence, which could be masked when looking at single features individually. In this section, we asked how such interaction can be studied if, instead of single-neuron perturbations, the interactome of the network is mapped by multiple-neuron perturbations. To this end, we investigated the effect of double-neuron perturbations, in which two neurons are perturbed simultaneously to assay their combined influence on postsynaptic targets (*SI Appendix*, Fig. S9*A*). We repeated similar experiments as outlined before (e.g., in Fig. 2) for such double-cell perturbations, and analyzed the influence as a function of the similarity of each influencee to both influencers (*SI Appendix*, Fig. S9*B*). Feature-specific suppression was evident for the moderate regime of RF similarity, especially for the region with moderate RF similarity to both influencers. Regions with the least RF similarity to one influencer, in contrast, revealed the most amplification when the RF similarity was high with regard to the other influencer (*SI Appendix*, Fig. S9*B*). Projecting the influence over a single dimension composed of both influencers revealed a stronger feature-specific suppression profile, when assayed as a function of RF similarity or an individual feature (preferred spatial phase) (*SI Appendix*, Fig. S9*C*).

The interaction of influencers thus confers more feature-specific suppression on average. This interaction can be more systematically studied by analyzing if the conjoint influence of two influencers is synergistic or antagonistic, namely whether the perturbation of neuron B in addition to neuron A increases or decreases their influence in isolation (*SI Appendix*, Fig. S9*D*). To analyze this, we developed a “synergy index,” which quantifies if the change in double-neuron influences is amplifying or suppressing the single-neuron effects (*SI Appendix*, *Methods*). The synergy index is computed for each A–B–C triplet, where A is the first influencer neuron, B is the second neuron that is additionally perturbed, and C is the target influencee of single- and double-neuron perturbations. The average synergy over all target influencees (Cs) for a sample influencer A and all other second influencers (Bs) is shown in *SI Appendix*, Fig. S9*E*, as a function of the response correlation of A and Bs. The average synergy reveals a net positive synergy for all A–B pairs, but this effect is more prominent for A–B pairs with high response correlations. Similar trends were observed when we calculated such average synergy curves for other example influencers (As) in the network (*SI Appendix*, Fig. S9*F*). These results suggest that double-neuron (and, more generally, multiple-neuron) perturbations can be employed in future experiments to map the perturbome of neuronal networks, by analyzing the synergy of their interactions.

### Functional Consequences for Sensory Processing.

**Fig. 6.**
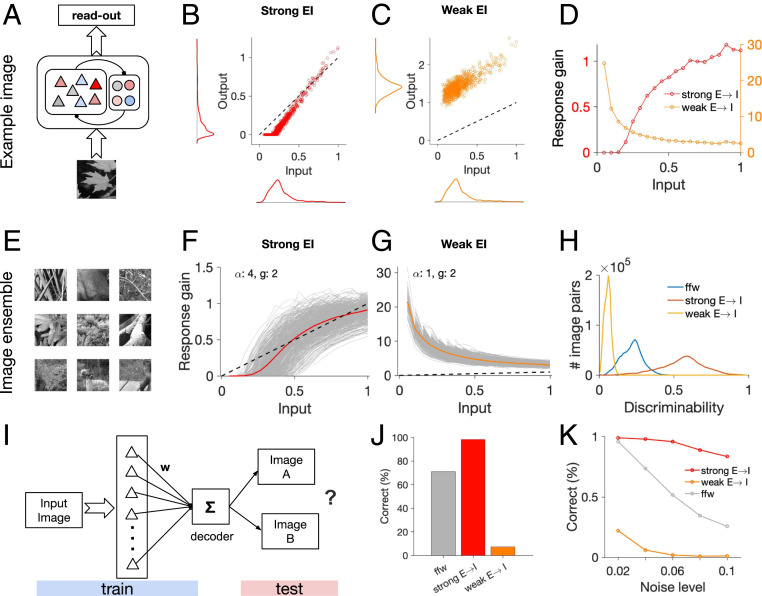
Population responses in different regimes and consequences for sensory processing. (*A*) The response of the network is obtained by projecting the input image over RF of neurons (feedforward input) and accounting for recurrent interactions resulting from the weight matrix. (*B*) Input and output of excitatory neurons in the network for a weight matrix with strong E–I interaction. Input and output are both normalized to the maximum level of input in response to the image. Distribution of input and output are shown on respective axes. (*C*) Same as *B* for a weight matrix with weak E→I. (*D*) Response gain (output divided by input) for each excitatory neuron as a function of input, for weight matrices with weak and strong E→I, respectively. (*E*) Sample images from an ensemble of 600 natural images used to test the networks. (*F*) Individual response gain curves (as in *D*) for individual images (gray) and their average across all images (red). (*G*) Same as in *F* for a weight matrix with weak E→I connections. (*H*) Discriminability of population responses calculated as the normalized angle (divided by 90°) of the vectors of population responses to all pairs of natural images (*SI Appendix*, *Methods*). (*I*) For each image, a decoder is trained to distinguish the image from half of the images in the ensemble, and then tested to distinguish the image from images in the other half. (*J*) Percentage of the correct responses of decoder in *I* in distinguishing the target image. (*K*) Percentage of correct responses at different levels of noise.

Our work so far revealed how different connectivity profiles lead to various patterns of feature-specific suppression/amplification when individual neurons are perturbed. However, single neurons are rarely perturbed in isolation. Instead, the realistic operating regime of the brain involves collective activation of neurons, for instance in response to external stimuli. To examine the functional consequences of such single-cell properties in naturalistic conditions, we therefore need to study the response of populations of neurons in different regimes. To address this, we presented natural images to large-scale visual networks. The feedforward input was obtained by filtering the images by the RF of individual neurons, and the output of the network was read from the population activity (Fig. 6*A*). We then analyzed how different networks transformed the input to output in different regimes.

We first looked at networks similar to those with feature-specific suppression/amplification as a result of single-neuron perturbations (e.g., as in Fig. 2). We analyzed the output activity of excitatory neurons as a function of their input in response to a sample image (Fig. 6*B*). For such networks, the activity of neurons with small inputs (small feedforward projections) was suppressed, while the activity of neurons with medium and large feedforward projections was mainly maintained or amplified (Fig. 6*B*). To understand what underlies such a transformation, we repeated the input–output analysis for the same network but with weaker E–I connectivity. Here, we did not observe the same nonlinear transfer function; instead, output responses were generally amplified with respect to the input (Fig. 6*C*).

To quantify the transformation, we calculated the response gain for each neuron as a factor with which the input needs to be multiplied to obtain the output. Neurons in the original networks (with strong E–I connections) showed a sigmoid response gain function: close to zero gains for small inputs and high gains for larger inputs, with a saturation trend at very high feedforward projections (Fig. 6*D*). In contrast, the network with weak E–I connections showed the opposite trend: higher gains for neurons with small feedforward projections, and the least amplification for intermediate and large inputs (Fig. 6*D*). We characterized such response gain curves for an ensemble of natural images (Fig. 6*E*) and observed similar nonlinear behavior for both networks across the images (Fig. 6 *F* and *G*). These results suggest that recurrent interactions in the networks with strong E–E connections can amplify the “noise” if the E–I interaction is weak; however, when strong and functionally specific connectivity exists between E–E and E–I connections, neuronal responses show a selective suppression of the noise and enhancement of the signal.

Lack of sigmoid-shape transfer functions in networks with weaker E–I connections can be a result of less inhibition in the network. We therefore asked whether an unselective increase of inhibition in our networks can compensate for the weakening of E–I connections, and restore the transfer function. To test that, we studied networks with an increase in inhibition dominance or broadness of the inhibitory connectivity, similar to the procedure we used before to evaluate different regimes of feature-specific suppression/amplification in these networks (cf. Fig. 3 *B* and *C*). Under both scenarios, we observed qualitatively similar response gain curves as in networks with weak E–I connections, and sigmoid nonlinearity did not emerge as a result of nonspecific inhibition (*SI Appendix*, Fig. S10). These results corroborate that strong and functionally specific E–I connections, which were necessary to obtain feature-specific suppression and amplification for different regimes, are also necessary for the emergence of sigmoid-shape nonlinear transfer functions, which can potentially enhance sensory coding.

To evaluate more directly the contribution of such nonlinear transfer functions to sensory processing, we studied the representation of natural images in different networks. We assessed how population responses to different images could be distinguished in networks with different nonlinearities. This was measured by quantifying the distance between population vectors in the *N*-dimensional space of neural activity (where *N* is the number of neurons). If the nonspecific component induced by all images over neurons is smaller than the specific projections, the distance between the vectors of population activity is small, and hence it is more difficult to discriminate different images. On the other hand, orthogonal representations, namely patterns of activity with the least overlap, enable the most discriminability for image pairs. We quantified such discriminability of population representations for all pairs of natural images in different networks (*SI Appendix*, *Methods*). The results revealed that networks with strong E–I connections increased the discriminability of feedforward projections, whereas networks with weak E–I had lower discriminability (Fig. 6*H*). By suppressing the redundant information and enhancing the representation of more informative neurons, networks with the sigmoid nonlinearity can therefore provide a more efficient population code to represent natural images.

The enhancement in the encoding of visual stimuli should lead to better decoding capacities of visual networks. To test this directly, we assessed the capacity of different networks in distinguishing different natural images. We trained a decoder to discriminate a target image from other images in the ensemble, based on the population activity of excitatory neurons (*SI Appendix*, *Methods*). We then tested the discrimination accuracy of the decoder when other nontarget images (not seen during the training) were shown, under different levels of noise (Fig. 6*I*). Decoders which performed their discrimination based on the population activity of networks with strong E–I connections performed significantly better than decoders based on the feedforward input, while networks with weak E–I weights did much worse than both (Fig. 6*J*). Increasing the level of noise reduced the accuracy for all networks, but networks with strong E–I connections outperformed other networks consistently; moreover, they showed the most robust behavior and were affected the least by noise (Fig. 6*K*). We therefore conclude that nonlinear response gains, emerging in networks with strong E–I connections and feature-specific suppression/amplification in single-neuron perturbations, improve image processing by increasing the capacity of a downstream decoder to distinguish different stimuli.

## Discussion

We presented computational models and mathematical analysis of the functional effects of single-neuron perturbations in neuronal networks. Our results revealed specific connectivity motifs necessary for the emergence of feature-specific suppression and amplification for moderately similar and highly similar RFs. Particularly, strong and specific E–I connections were necessary to explain the experimental results of single-neuron perturbations (5), and consistent with recent experimental reports of the specificity of E→I and I→E connections in the mouse visual cortex (12). Our theoretical analysis suggests that the most feature-specific suppression is achieved when these two motifs are balanced. Selective modulation of E→I and I→E connections in future experiments (e.g., by optogenetics techniques) can test individual contribution of different connectivity motifs to the influence, as predicted by the model.

We found that the same connectivity profiles also gave rise to the nonlinearity of neuronal responses underlying the ability to discriminate natural images. A similar nonlinearity has been suggested to explain the visual responses to natural images in mouse V1 (figure 2*G* in ref. 22). A prediction of our model is that such a sigmoid nonlinearity can be an emergent property of neuronal responses at the population level, as networks with different connectivity profiles expressed different nonlinearities in our simulations. Our model neurons in fact lacked such nonlinear transfer function at the single-cell level. Pyramidal neurons in the real cortex have a large dynamic range and show rather linear response curves (23), which can be a combined result of dendritic mechanisms (24) and operation in fluctuation-driven regimes of activity (25). It is therefore possible that such a nonlinearity in fact emerges at the population level and as a result of recurrent E–I interactions in the network. This prediction, and its potential link to the representation capacity of the population code, can thus be tested in future experiments by selective perturbation of the recurrent circuitry.

Strong E–I connectivity has been reported in many cortices across different species (13, 26, 27). Specifically, very large excitatory inputs from pyramidal cells to inhibitory neurons have been observed in humans, a property that has been absent in any other nonhuman cortices (26, 28), which may argue for the prominent role of this connectivity motif in complex cognitive processing. However, in the absence of specific mapping of their functional properties, and in view of the broad selectivity of inhibitory neurons (13, 29), it has been assumed that these connections are nonspecific and provide a blanket of inhibition to the local network (14, 18, 19). Recent studies, on the other hand, have revealed the emergence of specific E–I subnetworks, emphasizing the potential significance of selective inhibition for cortical computation (30–32). In mouse visual cortex, dendrite-targeting somatostatin-positive (SOM+) inhibitory neurons have been reported to have comparable levels of orientation selectivity to excitatory neurons in both L4 and L23 (21), and even within parvalbumin-positive (PV+) interneurons a range of selectivity has been observed depending on the extent of their dendritic tree (20). Future studies are thus needed to address the functional connectivity of E–I subnetworks more systematically to shed light on the specificity of E–I interactions. A case in point is a recent study of odor processing in the olfactory bulb of larval zebrafish (33). By mapping the functional connectomics via dense reconstructions of wiring diagrams (as opposed to sparse sampling of connections), the study could shed light on the higher-order interactions of excitatory and inhibitory neurons. Interestingly, the study found that bidirectional E–I connectivity is implicated in the decorrelation of odor responses via feature suppression.

Our work described how different connectivity patterns can lead to different perturbation effects. In networks with sparse E-to-E connectivity, we found that connected neurons primarily amplify each other upon single-neuron perturbations, while suppressive influences emerge via polysynaptic pathways. This may explain the experimental finding that neurons with higher noise correlations show higher influence, on average (5). The structure of noise correlation might, however, be governed by other parameters that we have not considered in our current model. For instance, it has been shown that balanced networks with spatially localized lateral projections can show high noise correlations (cf. figures 4 and 5 in ref. 34) compared to classic balanced networks with random connectivity (35–38). This was also shown in ref. 38, where distance-dependent connectivity profiles with localized excitation and global inhibition can generate higher noise correlations compared to networks with random connectivity (cf. figure 7 in ref. 38). The structure of noise correlations arising from these configurations may explain the pattern experimentally observed (cf. figure 3G in ref. 5). Note, however, that “distance”-dependent connectivity can also arise in the feature space, which can be the case in networks with sharper feature-specific connectivity of excitatory neurons, compared to inhibition.

In addition to linking connectivity and coding in single-neuron perturbations, our theory outlines how multiple-cell perturbations can be used to study functional properties of neuronal networks, by mapping higher-order interactions between influencers. A similar mathematical approach has been used recently to analyze the interaction of drugs and their resulting changes in cell morphologies, in order to shed light on the link between drug combinations and treatment of diseases (39). Targeted multiple-neuron perturbations of functionally identified neurons have in fact been used recently to shed light on the dynamics of persistent activity and short-term memory in mice (40). Similar approaches can be recruited to reveal how neurons work in tandem to shape functional processing in sensory cortices, with the possibility that different network perturbomes can dissociate between functional and dysfunctional circuitries (41, 42).

Our study also suggests that the temporal dynamics of the evolution of feature-specific suppression/amplification can reveal fundamental insights about the operation of the network. In our model neuronal networks, we found that feature-specific suppression emerges later than feature-specific amplification as a result of polysynaptic interactions (Fig. 4). Testing if such a pattern also exists in the cortex would have implications for the connectivity and function of cortical networks (e.g., transient responses versus sustained activity). Although technically challenging, future experiments can use population voltage-based measurements (43, 44) combined with single-neuron optogenetic perturbations to cast light on this important aspect. The temporal profile of functional influence can be further combined with multiple-neuron perturbations to map the temporal perturbome of neuronal networks, which will provide a more complete picture of functional and temporal patterns of processing in the brain.

In summary, our study provides a general mathematical framework to study the effect of single- (and multiple-) neuron perturbations in excitatory–inhibitory neuronal networks. By applying it to the visual cortex, we could unveil connectivity principles underlying the emergence of feature-specific influence in recent single-neuron perturbations and predict further properties of visual networks. The model specifically provided an explanation for the mutual presence of functionally specific excitatory connectivity and feature-specific suppressive influence of perturbations, thus reconciling previous experimental results (5–8). The modeling framework can be used in future studies to link cortical connectivity and dynamics to function in perturbation experiments.

## Methods

The activity of neuronal networks was simulated by solving the following:

$$\tau \frac{dr}{dt}=-r+{\left[Wr+s\right]}_{+},$$

where r is the vector of firing rates, W is the weight matrix, s is the external input, and τ is the time constant of integration. The weight of connection between neurons $\left(w_{ij}\right)$ was modulated as a function of RF similarity of neurons:

$$w_{ij}=J\exp\left(\text{η}\;CC_{ij}\right)+\text{ζ,}$$

where $CC_{ij}$ is the correlation coefficient of respective RFs, η determines the specificity of connections, and ζ is added noise. RFs were simulated as Gabor functions.

Influence (ψ) was assayed by perturbing the input to single or multiple neurons (δs), calculating the difference in the average activity of neurons as a result of perturbations (δr), and normalizing that by the size of input perturbation (δ*p*):

ψ=δr/δp.

Full experimental procedures are provided in *SI Appendix*, *Methods*. Codes for reproducing main simulations and results are freely available from ModelDB (http://modeldb.yale.edu/262045).

## Supplementary Material

Supplementary File

## Data Availability

All study data are included in the article and *SI Appendix*.

## References

[1] L. Fenno, O. Yizhar, K. Deisseroth, The development and application of optogenetics. Annu. Rev. Neurosci. 34, 389–412 (2011). 21692661 10.1146/annurev-neuro-061010-113817 PMC6699620

[2] O. Yizhar, L. E. Fenno, T. J. Davidson, M. Mogri, K. Deisseroth, Optogenetics in neural systems. Neuron 71, 9–34 (2011). 21745635 10.1016/j.neuron.2011.06.004

[3] E. S. Boyden, Optogenetics and the future of neuroscience. Nat. Neurosci. 18, 1200–1201 (2015). 26308980 10.1038/nn.4094

[4] A. M. Jose, The analysis of living systems can generate both knowledge and illusions. eLife 9, e56354 (2020). 32553111 10.7554/eLife.56354 PMC7302876

[5] S. N. Chettih, C. D. Harvey, Single-neuron perturbations reveal feature-specific competition in V1. Nature 567, 334–340 (2019). 30842660 10.1038/s41586-019-0997-6 PMC6682407

[6] H. Ko., The emergence of functional microcircuits in visual cortex. Nature 496, 96–100 (2013). 23552948 10.1038/nature12015 PMC4843961

[7] W.-C. A. Lee., Anatomy and function of an excitatory network in the visual cortex. Nature 532, 370–374 (2016). 27018655 10.1038/nature17192 PMC4844839

[8] L. Cossell., Functional organization of excitatory synaptic strength in primary visual cortex. Nature 518, 399–403 (2015). 25652823 10.1038/nature14182 PMC4843963

[9] H. Ko., Functional specificity of local synaptic connections in neocortical networks. Nature 473, 87–91 (2011). 21478872 10.1038/nature09880 PMC3089591

[10] A. D. Lien, M. Scanziani, Tuned thalamic excitation is amplified by visual cortical circuits. Nat. Neurosci. 16, 1315–1323 (2013). 23933748 10.1038/nn.3488 PMC3774518

[11] Y. T. Li, L. A. Ibrahim, B. H. Liu, L. I. Zhang, H. W. Tao, Linear transformation of thalamocortical input by intracortical excitation. Nat. Neurosci. 16, 1324–1330 (2013). 23933750 10.1038/nn.3494 PMC3855439

[12] P. Znamenskiy., Functional selectivity and specific connectivity of inhibitory neurons in primary visual cortex. bioRxiv:10.1101/294835 (4 April 2018).

[13] S. B. Hofer., Differential connectivity and response dynamics of excitatory and inhibitory neurons in visual cortex. Nat. Neurosci. 14, 1045–1052 (2011). 21765421 10.1038/nn.2876 PMC6370002

[14] A. M. Packer, R. Yuste, Dense, unspecific connectivity of neocortical parvalbumin-positive interneurons: A canonical microcircuit for inhibition? J. Neurosci. 31, 13260–13271 (2011). 21917809 10.1523/JNEUROSCI.3131-11.2011 PMC3178964

[15] C. K. Pfeffer, M. Xue, M. He, Z. J. Huang, M. Scanziani, Inhibition of inhibition in visual cortex: The logic of connections between molecularly distinct interneurons. Nat. Neurosci. 16, 1068–1076 (2013). 23817549 10.1038/nn.3446 PMC3729586

[16] J. Trousdale, Y. Hu, E. Shea-Brown, K. Josić, Impact of network structure and cellular response on spike time correlations. PLOS Comput. Biol. 8, e1002408 (2012). 22457608 10.1371/journal.pcbi.1002408 PMC3310711

[17] S. Sadeh, S. Rotter, Orientation selectivity in inhibition-dominated networks of spiking neurons: Effect of single neuron properties and network dynamics. PLOS Comput. Biol. 11, e1004045 (2015). 25569445 10.1371/journal.pcbi.1004045 PMC4287576

[18] E. Fino, R. Yuste, Dense inhibitory connectivity in neocortex. Neuron 69, 1188–1203 (2011). 21435562 10.1016/j.neuron.2011.02.025 PMC3086675

[19] M. M. Karnani, M. Agetsuma, R. Yuste, A blanket of inhibition: Functional inferences from dense inhibitory connectivity. Curr. Opin. Neurobiol. 26, 96–102 (2014). 24440415 10.1016/j.conb.2013.12.015 PMC4024353

[20] C. A. Runyan, M. Sur, Response selectivity is correlated to dendritic structure in parvalbumin-expressing inhibitory neurons in visual cortex. J. Neurosci. 33, 11724–11733 (2013). 23843539 10.1523/JNEUROSCI.2196-12.2013 PMC3724550

[21] W. P. Ma., Visual representations by cortical somatostatin inhibitory neurons—selective but with weak and delayed responses. J. Neurosci. 30, 14371–14379 (2010). 20980594 10.1523/JNEUROSCI.3248-10.2010 PMC3001391

[22] T. Yoshida, K. Ohki, Natural images are reliably represented by sparse and variable populations of neurons in visual cortex. Nat. Commun. 11, 872 (2020). 32054847 10.1038/s41467-020-14645-x PMC7018721

[23] S.-H. Lee., Activation of specific interneurons improves V1 feature selectivity and visual perception. Nature 488, 379–383 (2012). 22878719 10.1038/nature11312 PMC3422431

[24] S. L. Smith, I. T. Smith, T. Branco, M. Häusser, Dendritic spikes enhance stimulus selectivity in cortical neurons in vivo. Nature 503, 115–120 (2013). 24162850 10.1038/nature12600 PMC6319606

[25] C. van Vreeswijk, H. Sompolinsky, Chaos in neuronal networks with balanced excitatory and inhibitory activity. Science 274, 1724–1726 (1996). 8939866 10.1126/science.274.5293.1724

[26] G. Molnár., Complex events initiated by individual spikes in the human cerebral cortex. PLoS Biol. 6, e222 (2008). 18767905 10.1371/journal.pbio.0060222 PMC2528052

[27] G. Molnár., Human pyramidal to interneuron synapses are mediated by multi-vesicular release and multiple docked vesicles. eLife 5, e18167 (2016). 27536876 10.7554/eLife.18167 PMC4999310

[28] V. Szegedi., Plasticity in single axon glutamatergic connection to GABAergic interneurons regulates complex events in the human neocortex. PLoS Biol. 14, e2000237 (2016). 27828957 10.1371/journal.pbio.2000237 PMC5102409

[29] D. D. Bock., Network anatomy and in vivo physiology of visual cortical neurons. Nature 471, 177–182 (2011). 21390124 10.1038/nature09802 PMC3095821

[30] A. G. Khan., Distinct learning-induced changes in stimulus selectivity and interactions of GABAergic interneuron classes in visual cortex. Nat. Neurosci. 21, 851–859 (2018). 29786081 10.1038/s41593-018-0143-z PMC6390950

[31] F. Najafi., Excitatory and inhibitory subnetworks are equally selective during decision-making and emerge simultaneously during learning. Neuron 105, 165–179.e8 (2020). 31753580 10.1016/j.neuron.2019.09.045 PMC6952547

[32] D. E. Wilson., GABAergic neurons in ferret visual cortex participate in functionally specific networks. Neuron 93, 1058–1065.e4 (2017). 28279352 10.1016/j.neuron.2017.02.035 PMC5477844

[33] A. A. Wanner, R. W. Friedrich, Whitening of odor representations by the wiring diagram of the olfactory bulb. Nat. Neurosci. 23, 433–442 (2020). 31959937 10.1038/s41593-019-0576-z PMC7101160

[34] R. Rosenbaum, M. A. Smith, A. Kohn, J. E. Rubin, B. Doiron, The spatial structure of correlated neuronal variability. Nat. Neurosci. 20, 107–114 (2017). 27798630 10.1038/nn.4433 PMC5191923

[35] C. van Vreeswijk, H. Sompolinsky, Chaotic balanced state in a model of cortical circuits. Neural Comput. 10, 1321–1371 (1998). 9698348 10.1162/089976698300017214

[36] A. Renart., The asynchronous state in cortical circuits. Science 327, 587–590 (2010). 20110507 10.1126/science.1179850 PMC2861483

[37] T. Tetzlaff, M. Helias, G. T. Einevoll, M. Diesmann, Decorrelation of neural-network activity by inhibitory feedback. PLOS Comput. Biol. 8, e1002596 (2012). 23133368 10.1371/journal.pcbi.1002596 PMC3487539

[38] V. Pernice, B. Staude, S. Cardanobile, S. Rotter, How structure determines correlations in neuronal networks. PLOS Comput. Biol. 7, e1002059 (2011). 21625580 10.1371/journal.pcbi.1002059 PMC3098224

[39] M. Caldera., Mapping the perturbome network of cellular perturbations. Nat. Commun. 10, 5140 (2019). 31723137 10.1038/s41467-019-13058-9 PMC6853941

[40] K. Daie, K. Svoboda, S. Druckmann, Targeted photostimulation uncovers circuit motifs supporting short-term memory. bioRxiv:10.1101/623785 (30 April 2019). 10.1038/s41593-020-00776-3 33495637

[41] R. A. Ozdemir., Individualized perturbation of the human connectome reveals reproducible biomarkers of network dynamics relevant to cognition. Proc. Natl. Acad. Sci. U.S.A. 117, 8115–8125 (2020). 32193345 10.1073/pnas.1911240117 PMC7149310

[42] S. Qiao, J. I. Sedillo, K. A. Brown, B. Ferrentino, B. Pesaran, A causal network analysis of neuromodulation in the mood processing network. Neuron 107, 972–985.e6 (2020). 32645299 10.1016/j.neuron.2020.06.012 PMC7486259

[43] K. D. Piatkevich., Population imaging of neural activity in awake behaving mice. Nature 574, 413–417 (2019). 31597963 10.1038/s41586-019-1641-1 PMC6858559

[44] T. Knöpfel, C. Song, Optical voltage imaging in neurons: Moving from technology development to practical tool. Nat. Rev. Neurosci. 20, 719–727 (2019). 31705060 10.1038/s41583-019-0231-4

