# Supplementary material for: Theory of neuronal perturbome in cortical networks
Source: Proc Natl Acad Sci U S A. 2020 Oct 14;117(43):26966–76. doi: 10.1073/pnas.2004568117 (PMC7604497; doi:10.1073/pnas.2004568117)
Supplement: Supplementary File [file pnas.2004568117.sapp.pdf]

Supplementary Information for

Theory of neuronal perturbome in cortical networks

Sadra Sadeh and Claudia Clopath

For correspondence: Claudia Clopath
Email: c.clopath@imperial.ac.uk

This PDF file includes:

Figures S1 to S10

Supplementary text (Methods)

www.pnas.org/cgi/doi/10.1073/pnas.2004568117

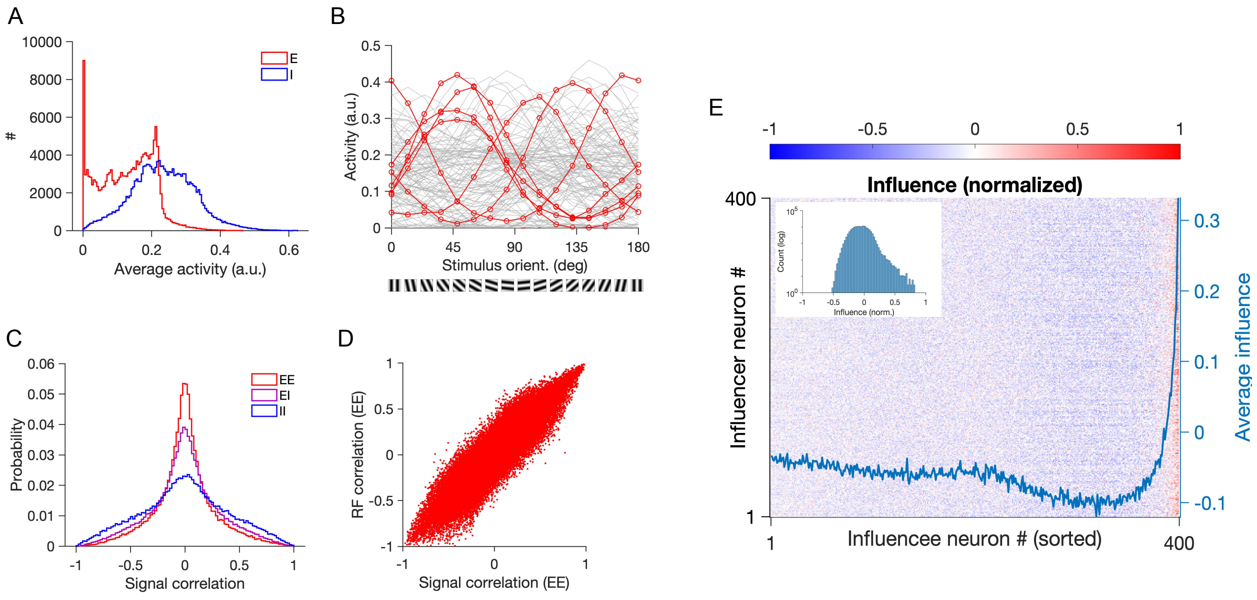

Fig. S1. Response statistics and distribution of influence for all neurons in the network. **(A)** Distribution of firing rates of 400 excitatory (E) and 400 inhibitory (I) neurons in the network of **Fig. 2**, in response to 400 static gratings with different orientations, spatial frequencies and spatial phases. The average response of each neuron to each stimulus is calculated during the stationary state after the initial transient response (i.e. from T = 100 to T = 500 after stimulus onset). **(B)** Sample tuning curves of E neurons in the network in response to 16 gratings with different orientations (0 to 180 degrees), but with a fixed spatial frequency (SF = 0.04) and spatial phase (PH = 0º), shown on the bottom. Gray curves show the tuning curves of 100 randomly chosen neurons, and the red curves are 7 samples from neurons with closest preferred SFs and phases to the gratings (0.02<SF<0.06 and -45º<PH<45º). **(C)** Distribution of signal correlations between all excitatory-excitatory (EE), excitatory-inhibitory (EI) and inhibitory-inhibitory (II) pairs. Signal correlation is calculated as the correlation coefficient of the vectors containing the average responses of neurons to stimuli described in (A). **(D)** Relationship between signal correlations as described in (C) with correlation of RFs for all EE pairs. **(E)** The matrix of normalized influence between influencers (different rows) and influencees (different columns), similar to **Fig. 2G** but for all excitatory neurons in the network. Influence is normalized by the maximum absolute value of influence between all excitatory pairs. In each row, the influencees are sorted according to their response correlation with the respective influencer in an ascending order. The average influence for each column is plotted on the right. Inset: The distribution of normalized influence for all excitatory pairs in the network. Note the logarithmic scale on the y-axis.

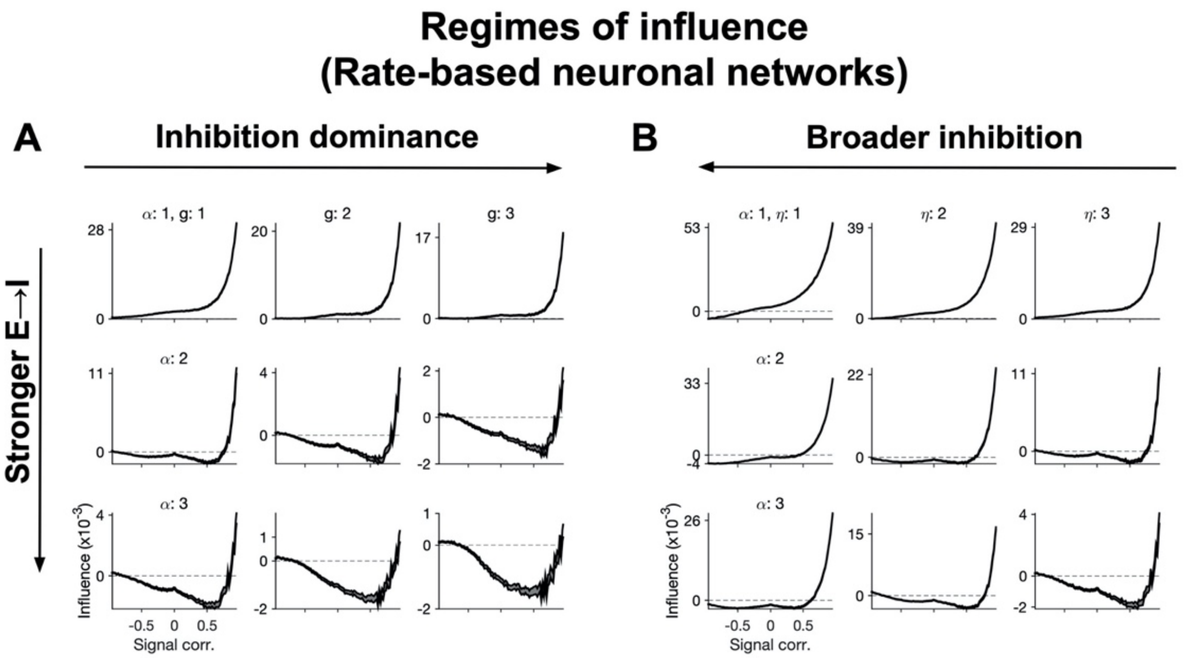

Fig. S2: **Regimes of feature-specific influence in rate-based neuronal networks.**

(A,B) Same as **Fig. 3B,C**, respectively, for rate-based neuronal networks with similar weight matrices. For each network, the weight matrix is generated with the same combination of parameters as in **Fig. 3B,C**, but instead of inferring the influence from the weight matrix (as in SI Appendix, **Methods**, **Eq. 19**), the influence is calculated from rate-based simulations of the network (SI Appendix, **Methods, Eq. 14**).

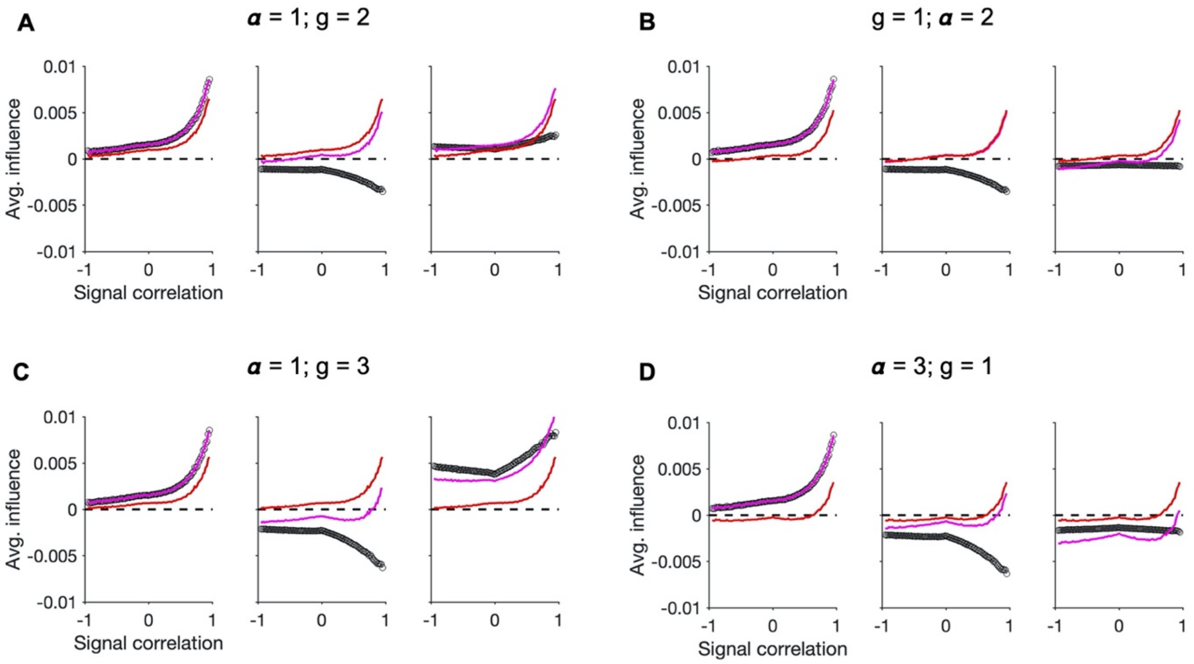

Fig. S3: Contribution of different influence motifs in different regimes of connectivity.
(A) Same as **Fig. 4A**, with weaker E→I ($\alpha = 1$), while other parameters remain the same (including g = 2). **(B)** Same as (A), with weaker I→{E,I} (g=1), while other parameters remain the same (including $\alpha = 2$). **(C)** Same as (A), but with increasing g to 3 to compensate for the relative weakness of E→I connections ($\alpha = 1$). **(D)** Same as (B), but with increasing α to 3 to compensate for weak inhibitory connections (g = 1).

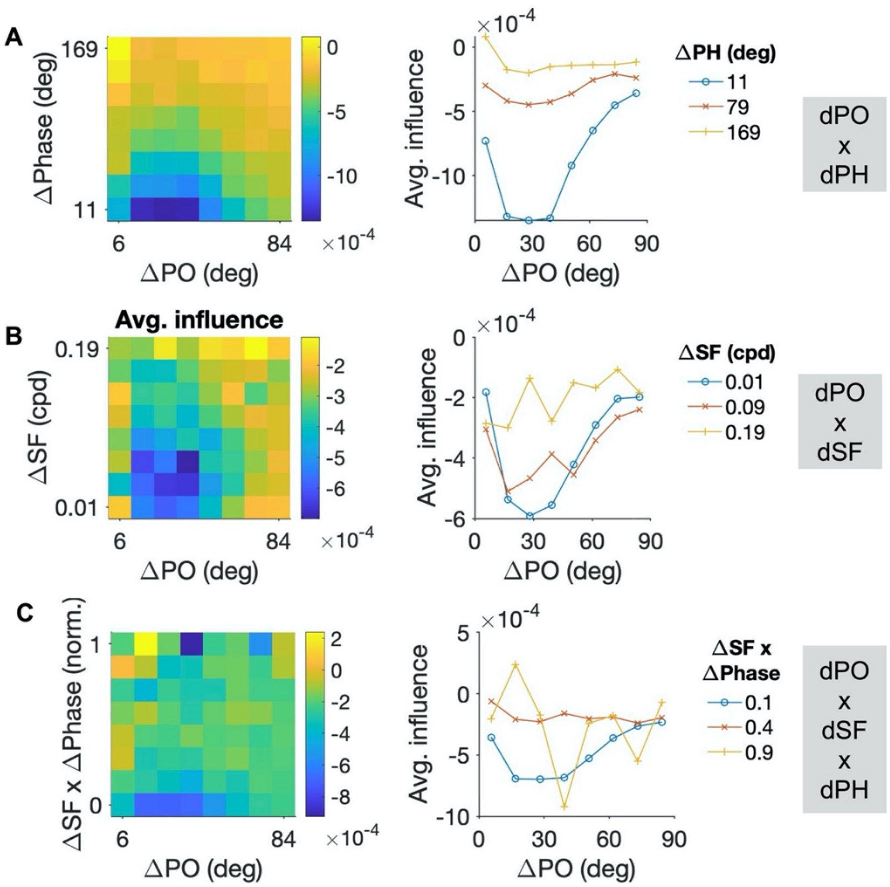

Fig. S4: Influence as a function of the interaction of individual features.

Same as **Fig. 5**, but for interaction of individual features. **(A)** Left: Average influence for all pairs within a given range of dPO and dPH. Right: Average influence as a function of dPO for three levels (minimum, medium and maximum) of dPH. **(B)** Same as (A) for the interaction of dPO and dSF. **(C)** Same as (A,B) for the interaction of dPO with the conjoint change of SF and phase (dSF x dPhase). dSF and dPhase are both normalized to their maximum values, respectively, and then multiplied to obtain a single variable.

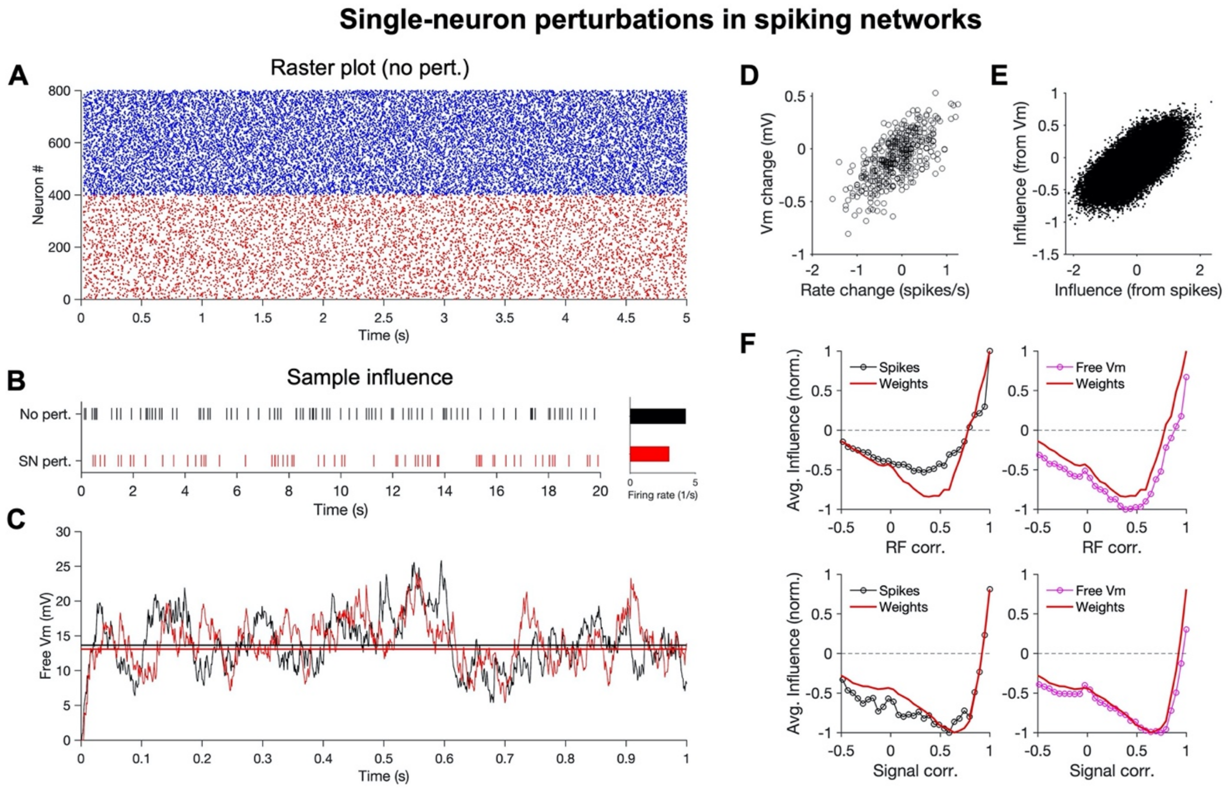

Fig. S5: Neuronal influence in spiking networks.

(A) Example raster plot of the spiking network composed of 400 E (red) and 400 I (blue) neurons, for 5 seconds of simulation in the baseline (absent perturbations). **(B)** Spiking activity of an example E neuron (influencee) before (No pert.) and after (SN pert.) a single-neuron perturbation, for the total duration of simulation (20 s). The neuron is suppressed, as shown by its average firing rate (right). **(C)** Free membrane potential (Vm) of the same neuron in (B), for 1 second of simulation, before (black) and after (red) perturbation. Lines show the average free Vm, respectively, during the total simulation time (20 s). Free Vm for each neuron was obtained by simulating an extra neuron with exactly the same inputs and dynamics but without the spiking mechanism and measuring its membrane potential. This provides the true estimate of the membrane potential in the absence of distortions induced by the spiking mechanism. **(D)** Relationship between the rate change of E neurons (influencees) in the network with the change in their free Vm, as a result of the perturbation of a sample E neuron (influencer). **(E)** Influence inferred from free Vm versus influence inferred from the spiking activity, for all pairs of E influencers and influencees. The influence is obtained as the change in the free Vm or the firing rate as a result of a single neuron perturbation, normalized by the size of perturbation. **(F)** Average influence as a function of RF correlations (top) or signal correlations (bottom), as inferred from spikes (left) or free Vm (right), compared with the prediction from the weight matrix (red).

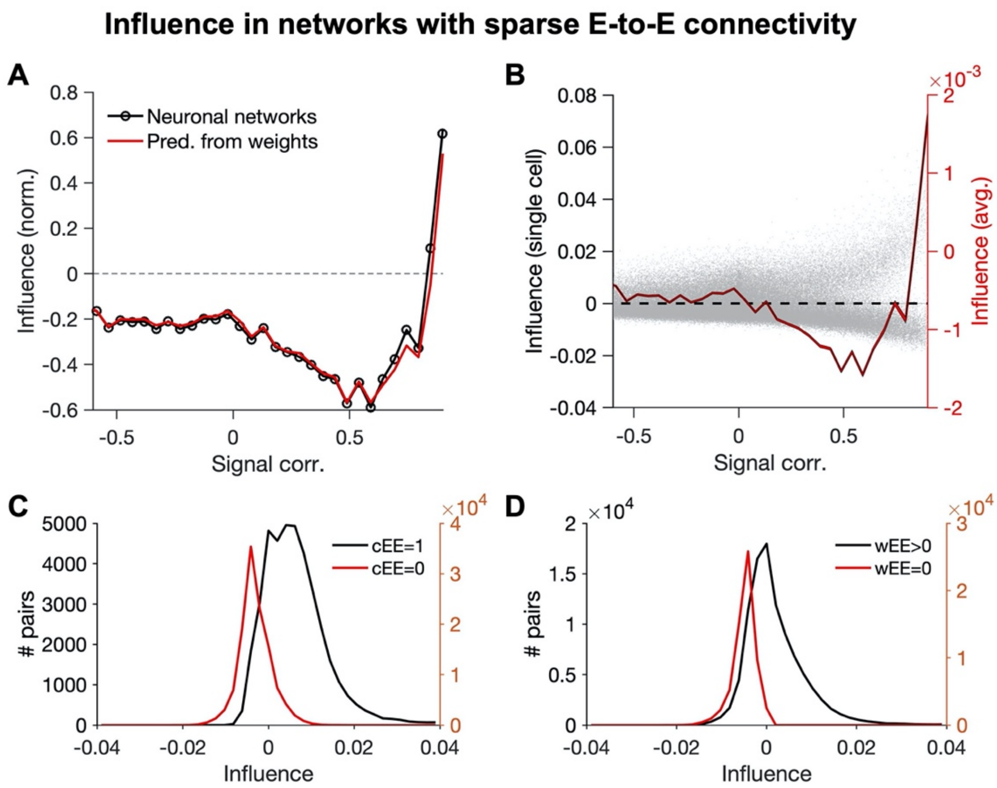

Fig. S6: Influence in networks with sparse EE connectivity.

(A) Average influence as a function of signal correlations in rate-based networks with sparse EE connectivity (connection probability: 25%). Connectivity between a pair of E neurons is obtained as $w_{ij} = J\, exp(\eta\, CC_{ij})c_{ij} + \zeta$, where c_{ij} is the initial connectivity from neuron j to i (c=1, connected; c=0, disconnected) and CC_{ij} is the RF similarity of the neurons. $J_{EE} = 0.01$ and ζ is a random value drawn from a normal distribution with $N(0{,}0.005)$. Weights smaller than 0 are set to zero to ensure non-negativity of excitatory weights. Influence inferred from average rates (black) are compared with the prediction from the weight matrix (red). **(B)** Influence for all pairs of E influencers and influencees (gray dots), compared with the grand average (red). **(C)** Distribution of influence for EE pairs with c = 1 (red) and c = 0 (black), where c is the initial connectivity parameter as described in (A). When $c_{ij} = 1$ (i.e. a connection exists between a presynaptic neuron j and a postsynaptic neuron i), the weight of synapse (w_{ij}) is modulated according to RF similarity of the pair of neurons. **(D)** Distribution of influence for EE pairs, when connectivity is assayed from actual synaptic weights. Neuron j is considered to be connected to neuron i, if $w_{ij} > 0$.

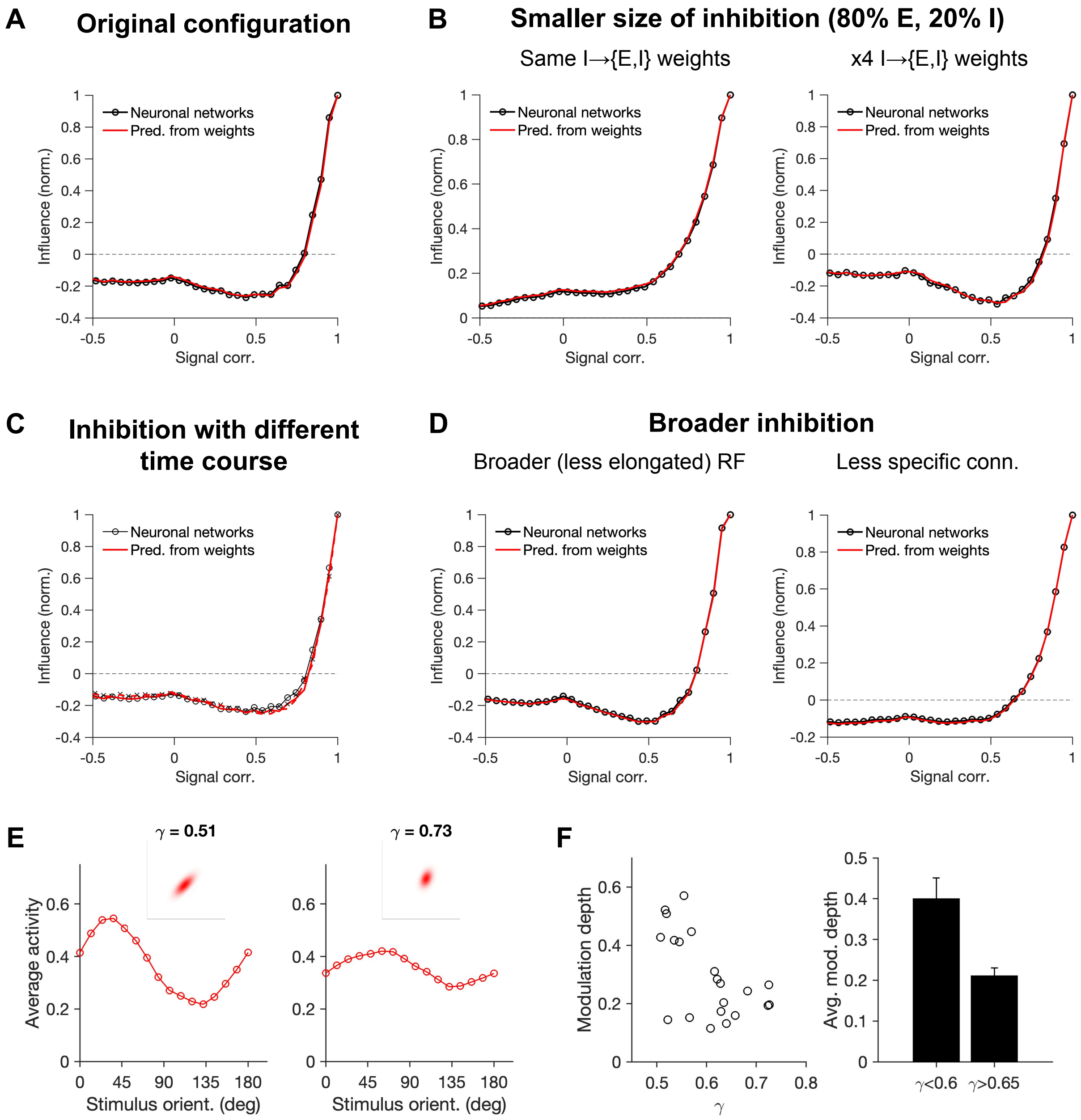

Fig. S7: Neuronal influence in networks with biologically realistic inhibition.

(A) Average total influence as a function of signal correlation in the original connectivity configuration of the network (g = 2, $\alpha = 2$, other parameters the same as **Fig. 2**). **(B)** Same as (A), for networks with smaller size of the inhibitory subpopulation (NE=800; NI = 200). Left: With the same I→{E,I} weights as before. Right: When I→{E,I} weights are increased 4 times, to compensate for less inhibitory neurons. **(C)** Same as (A), for simulations with slower or dynamics of inhibition: $\tau_I = 20$. $\tau_E = 10$ as before. The result of a simulation with faster dynamics of inhibition ($\tau_I = 5$) is shown for comparison (neuronal networks: black crosses; prediction from

weights: red dashed lines). **(D)** Same as (A), for networks with broader inhibition. Left: Results of simulations with less elongated RFs of inhibition: γ is drawn from a range between 0.5 and 0.75 for inhibitory neurons. $\gamma = 0.5$ for E neurons, as before. Right: Simulations with broader connectivity of inhibitory neurons. This is controlled by a reduction in the specificity of I→{E,I} and E→I connections, by allowing $\eta_{E\to I} = \eta_{I\to E} = \eta_{I\to I} = 1.75$. Other parameters, including the specificity of E→E weights ($\eta_{E\to E} = 2$), remain the same as before. **(E)** Two sample inhibitory RFs from networks in (D), left, with different degrees of elongation, as specified by the parameter γ. The tuning curve of each neuron is shown, when the network is stimulated with 16 gratings of different orientations (ranging from 0 to 180º), at a fixed spatial frequency (ω = 0.04) and spatial phase of ϕ= 0 º (same stimuli as in **Fig. S1B**). Sample RFs are chosen from inhibitory neurons with close SF (ω) and spatial phase (ϕ) to the gratings, with the criteria: $0.03 < \omega < 0.05$ and $-\pi/4 < \phi < \pi/4$. **(F)** Left: Modulation depth of tuning curves of all inhibitory neurons satisfying the previous criteria ($0.03 < \omega < 0.05$ and $-\pi/4 < \phi < \pi/4$) versus their respective RF elongation parameter (γ). Modulation depth is calculated as $(r_{max} - r_{min})/(r_{max} + r_{min})$, where r_{max} and r_{min} are the maximum and minimum activity in response to gratings. Right: Average modulation depth for the previous inhibitory neurons when broken to two different categories of elongated ($0.5 < \gamma < 0.6$) and less elongated ($0.65 < \gamma < 0.75$) RFs. Error bars show sem.

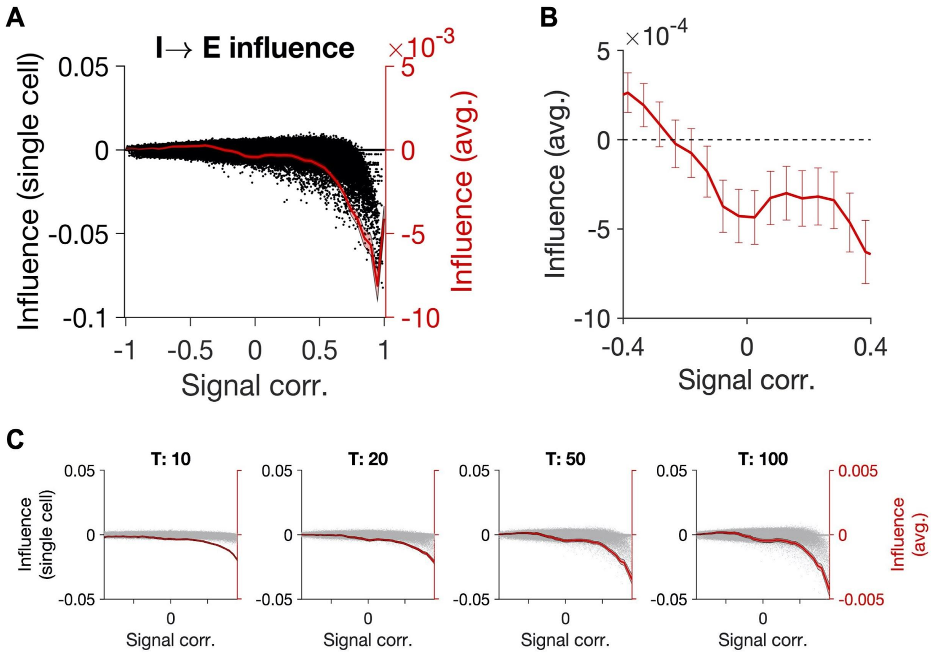

Fig. S8: **Influence inferred from perturbing single inhibitory neurons.**

(A) Influence of inhibitory neurons on excitatory neurons for all pairs (black) and as average calculated for all pairs within a given range of signal correlation (bin size: 0.05). **(B)** Zoom in for the intermediate range. Error bars denote ±sem. **(C)** Same as in **Fig. 4B** for inhibitory single-neuron perturbations.

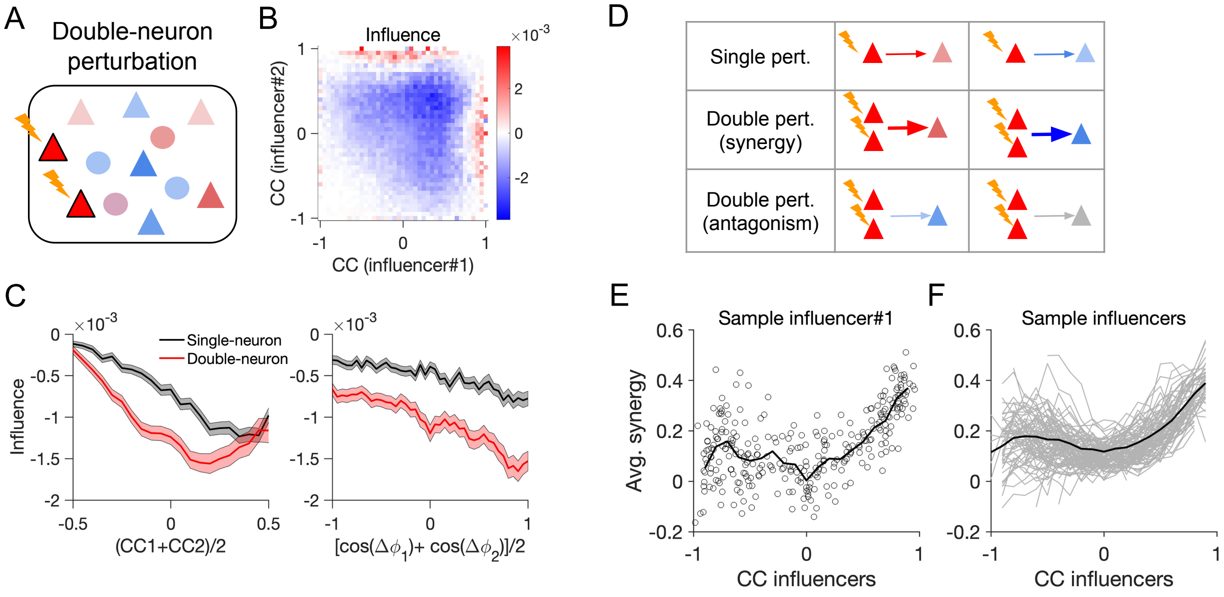

Fig. S9: Influence in double-cell perturbations.

(A) Double-cell influence is assayed in neuronal networks by perturbing two excitatory neurons and quantifying the result of this dual perturbation on other neurons in the network. **(B)** Average influence as a function of the average response correlation of the two influencers with all the influencees. **(C)** Left: Average influence as a function of the average response correlation of the two influencers with influencees in double-neuron perturbations (red). CC1=CC2 for single-neuron perturbations (black). Right: Average influence as a function of the average difference in the spatial phases of the influencers with the influencees. Cosine of dPHs is used to obtain a normalized measure between -1 (most dissimilar) to 1 (most similar). **(D)** Illustration of different outcomes of double-neuron perturbations compared to single-neuron results. First column: An influencer with a net positive influence on an influencee (first row) can experience synergistic interaction with another influencer if the net influence of the double-neuron perturbation is more positive (second row), or an antagonistic interaction if the net influence is less positive or negative (third row). Second column: Example of synergistic or antagonistic interaction for a negative single-neuron influence. **(E)** Average synergy index (see SI Appendix, **Methods**) of an example first influencer with all other influencees as a function of response correlation of the first influencer with the second influencers. Black line shows the average in each CC bin (bin width: 0.1). **(F)** The average synergy as a function of response correlation (black line in (E)) for 100 sample first influencers (gray). Black line shows the average across all curves. Bin width: 0.1.

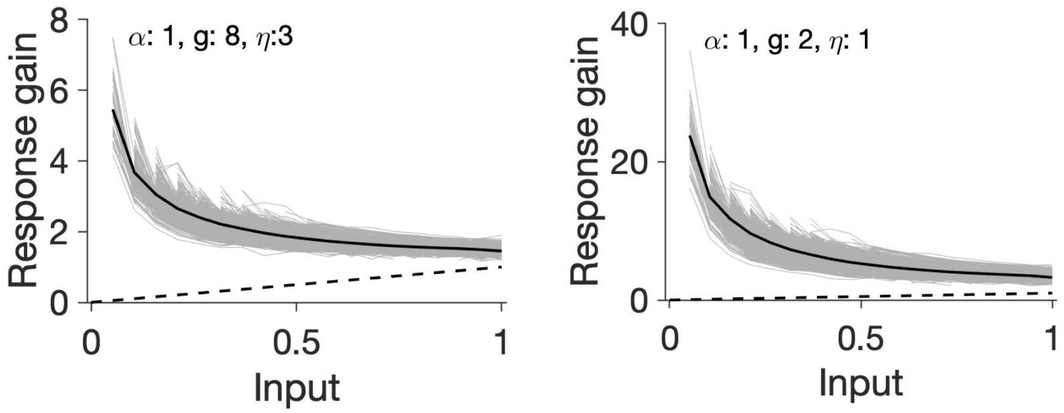

Fig. S10: **Response gain for networks with weak E→I and stronger or broader inhibition.** Same as **Fig. 7G**, for control conditions where the network with weak E→I connections had either higher inhibition dominance (left) or broader inhibition (right). Neither of the two conditions resulted in sigmoid nonlinearity of gains as observed in **Fig. 7F**.

Methods

1 Network simulations

1.1 Neuronal receptive fields

Visual receptive field (RF) of neurons were modeled as two-dimensional Gabor fields:

$$g_{\lambda,\theta,\phi,\sigma,\gamma}(x,y) = \exp(-\frac{x'^2+\gamma^2 y'^2}{2\sigma^2})\cos(2\pi\frac{x'}{\lambda}+\phi) \tag{1}$$

where

$$x' = x\cos(\theta) + y\sin(\theta)$$
$$y' = -x\sin(\theta) + y\cos(\theta).$$

x and y are the position on the visual field, σ sets the size, θ is the preferred orientation, $\omega = 1/\lambda$ the spatial frequency, and ϕ the spatial phase of the receptive field. RFs are instantiated on a $L \times L$ field, with their resolutions expressed in pixels per degree (ppd), and centered at (x_0, y_0). Unless stated otherwise, parameters are chosen as the default values below: $L = 50$, ppd = 4, $\sigma = 2.5$, $\gamma = 0.5$. The following parameters are drawn randomly from a uniform distribution: (x_0, y_0) from $[-1.25, 1.25]$ degrees, θ and ϕ from $[0, \pi)$ and $[0, 2\pi)$, respectively. Spatial frequency, ω, is drawn from a gamma distribution with shape parameter 2 and scale parameter 0.04 and 0.02 for excitatory and inhibitory neurons, respectively.

1.2 Neuronal connectivity

Network connectivity is represented by the weight matrix W, with the entry w_{ij} denoting the weight of the connection from presynaptic neuron j to postsynaptic neuron i. Connectivity is all-to-all and weights between two neurons are modulated as a function of similarity of their respective receptive fields. Functional similarity is assayed in two ways: first, by calculating

the correlation coefficient of the receptive fields (RF CC) directly:

$$\mathrm{CC}_{\mathrm{ij}}^{\mathrm{RF}} = \frac{\mathrm{cov}(\mathrm{g_i}, \mathrm{g_j})}{\sigma_{\mathrm{g_i}}\, \sigma_{\mathrm{g_j}}}. \tag{2}$$

Alternatively, functional similarity is assayed by using external stimuli. Consider a sequence of stimuli, e.g. N_{stim} static gratings or natural images. The functional behaviour of neuron i in response to this stimulus set can be described by a response vector ρ_i (with size N_{stim}). The k-th element of the vector represents the correlation coefficient of the neuronal RF with the k-th stimulus in the sequence. For a pair of neurons (i, j), the Response CC (also referred to as signal CC) is then calculated from the correlation of their response vectors, (ρ_i, ρ_j):

$$\mathrm{CC}_{\mathrm{ij}}^{\mathrm{response}} = \frac{\mathrm{cov}(\rho_{\mathrm{i}}, \rho_{\mathrm{j}})}{\sigma_{\rho_{\mathrm{i}}}\, \sigma_{\rho_{\mathrm{j}}}} \tag{3}$$

To evaluate response CCs in our simulations, we used 1000 static gratings with random preferred orientations between $[0, \pi]$ and spatial frequencies drawn from a gamma distribution with shape parameter 2 and scale parameter 0.04. Gratings were instantiated in the same fashion as the Gabor RFs described above, with the difference that they were extended in space to obtain full-field gratings. This was achieved by choosing very large values of σ in Eq. (1).

For each pair of neurons i and j, the weight between them is then modulated as a function of the respective measure of similarity ($\mathrm{CC_{ij}}$):

$$w_{ij} = J \exp(\eta\, \mathrm{CC_{ij}}) + \zeta \tag{4}$$

where $\mathrm{CC_{ij}}$ can be $\mathrm{CC}_{\mathrm{ij}}^{\mathrm{RF}}$ or $\mathrm{CC}_{\mathrm{ij}}^{\mathrm{response}}$ (this is specified in each case in the details of the simulation). J parameterises the strength of the respective weights (denoted as J_{XY}, where $\{X, Y\} \in \{E, I\}$), and η determines the sharpness of the exponential dependence of weights on similarity (with a default value of $\eta = 3$). ζ is an i.i.d distributed randomly chosen value between $[-\zeta_{max}, \zeta_{max}]$ added to each element, with $\zeta_{max} = 0.005$. For $E \to \{E, I\}$ weights the values smaller than 0, and for $I \to \{E, I\}$ weights values larger than 0, are clipped to 0. For a given value of $E \to E$ weights (J_{EE}), inhibition-dominance is parameterized by the relative surplus of $I \to \{E, I\}$ weights: $J_{IE} = J_{II} = -gJ_{EE}$. The relative strength of $E \to I$ weights are quantified by a similarly defined parameter, α: $J_{EI} = \alpha J_{EE}$. Broadening of inhibitory connectivity (e.g. in **Fig. 3C**) is controlled by changing the sharpness of the profile of $I \to \{E, I\}$ connections, by keeping η the same for other connections and changing η_{IE} and η_{II} specifically.

1.3 Neuronal simulations

The activity of neuronal networks was simulated by numerically solving the following equations:

$$\tau \frac{dr}{dt} = -r + [Wr + s]_+ \tag{5}$$

where r is a vector of all firing rates (for N neurons, composed of N_E excitatory and N_I inhibitory neurons), W is the matrix of connection weights as described above, s is the vector of external input to all neurons, τ is the effective time constant of integration, and $[]_+$ denotes half-wave rectification.

The influence is assayed by simulating the response of the network for a given time (T) in the baseline state (s_0) and with an extra perturbation of a single neuron: $s_p = s0 + \delta s_i$, where δs_i is a perturbation vector containing δp (the size of perturbation) for the i-th element and zeros for the rest of neurons. The average (temporal) firing rate of the neurons in the stationary state (after discarding the initial transient response for T_{trans}) is calculated for the baseline and the perturbed state as r_0 and r_p, respectively. The vector of change in firing rates, $\delta r = r_p - r_0$, is normalized by the size of perturbation to obtain the influence of neuron i (the influencer) on the rest of the network (influencees): $\psi = \delta r / \delta p$.

For double-neuron perturbations, the same procedure was repeated with the only difference that the vector of perturbations contained two non-zero elements for the two influencers with size δp, and influence was obtained by normalizing the induced changes in the activity of influencees by δp. Default values for the quantification of influence are: $N_E = N_I = 400$, $J = 0.0025$, $\tau = 10$, $T = 500$, $T_{\text{trans}} = 50$, $\delta p = 0.1$.

Influence in spiking networks was obtained by modelling the equations describing the membrane potential dynamics of leaky integrate-and-fire neurons:

$$\tau_m dV_m/dt \;=\; -V_m(t) + s(t) \tag{6}$$

where V_m denotes the membrane potential of a neuron, and $\tau_m = RC$ is the time constant of integration of the membrane potential, with R and C being the membrane resistance and capacitance, respectively. When the membrane potential reaches a voltage threshold, $V_{th} = 20\ mV$, a spike is elicited and the membrane potential is reset to the reset voltage, $V_{reset} = 0$. The input to the neuron at each time is described by $s(t) = R\ I(t)$, which comprises external input and internally generated input from recurrent activity. Once a spike is emitted in a presynaptic source, an instantaneous change in the membrane potential of all postsynaptic sources is emulated in the next simulation time step. The total input at time t for a postsynaptic neuron i can be expressed as $s(t) = \Sigma_j w_{ij} \delta_j(t)$, where $\delta_j(t)$ denotes the presence (1) or

absence (0) of spike in presynaptic sources (including external input or input from presynaptic neurons in the recurrent network), and w_{ij} describes the weight of connection (in mV) from the j-th presynaptic source. The weight matrix was generated in the same fashion as previous weight matrices, except weights were multiplied by V_{th}, such that the normalized weights (by threshold) were similar between the spiking and rate-based networks.

1.4 Data analysis

To analyse the behaviour of the influence as a function of signal correlation between influencers and influencees in different regimes (**Fig. 3**), we employed a feature-specific suppression/amplification (S/A) index. It was calculated from the average influence, which was obtained as the average influence between all pairs of influencers and influencees with signal correlations in a certain bin (with bin widths of 0.02) between -1 and 1. The index is composed of three submetrics: (1) x: the mean average influence in the intermediate regime; (2) y: the slope of the dependence of the average influence on signal correlation in the intermediate regime; and (3) z: the mean level of average influence in highly similar regime. The intermediate regime was defined as signal correlations between -0.3 to 0.3, and a highly similar regime was taken as the range of signal correlation between 0.7 to 0.9. Each submetric was normalized to the maximum of the absolute values for all the network simulations with different parameters tested (e.g. as in **Fig. 3B-G**). The feature-specific S/A index (SAI) was then obtained as:

$$\mathrm{SAI} = -\mathrm{x}/(|\mathrm{x}|) - \mathrm{y}/(|\mathrm{y}|) + \mathrm{z}/(|\mathrm{z}|) \tag{7}$$

The more the suppression at intermediate regimes *and* the more the amplification at highly similar regimes, the higher the SAI would be.

To quantify the combined influence of perturbing two influencers in double-neuron perturbations (**Fig. 6**), we developed a synergy index. For the first influencer (neuron i), the effect of additional perturbation of a second influencer (neuron j) on the influencee (neuron k) was quantified as follows:

$$\Delta\psi(i, j, k) = \psi(\{i, j\} \to k) - \psi(i \to k), \tag{8}$$

where $\psi(\{i, j\} \to k)$ is the influence of double-neuron perturbations of $\{i, j\}$ on k and $\psi(i \to k)$ is the single-neuron influence of i on k. The synergy of influence between the triplet $\{i, j, k\}$ was then calculated as:

$$\mathrm{syn}(\mathrm{i}, \mathrm{j}, \mathrm{k}) = \Delta\psi(\mathrm{i}, \mathrm{j}, \mathrm{k})/\psi(\mathrm{i} \to \mathrm{k}). \tag{9}$$

We excluded pairs with very small single-neuron perturbations ($\psi(i \to k) < 0.001$) to avoid their overrepresentation in the metric. The average synergy between two influencers (i, j) was

calculated by computing the mean synergy across all target influencees:

$$\mathrm{syn(i,j)} = \frac{1}{\mathrm{N}-2}\sum_{\mathrm{k}=1}^{\mathrm{N}-2}\mathrm{syn(i,j,k)} \tag{10}$$

Note that the synergy will be positive (synergistic) if the change in the influence as a result of the interaction of the second influencer is in the same direction as the original, single-neuron perturbation, and negative (antagonistic) otherwise. Thus, both suppression and amplification of single-neuron influences can undergo synergy (or antagonism) as a result of double-neuron perturbations, depending on whether the interaction exacerbates (or diminishes) the initial influence in the same (or reverse) direction.

1.5 Decoding of natural images

To evaluate the population responses of neuronal networks to external stimuli, we presented natural images to our model networks. Natural images were chosen from the McGill calibrated colour image database (http://tabby.vision.mcgill.ca). The feedforward input (I_{ffw}) to each neuron in response to each natural image was calculated as:

$$I_{\mathrm{ffw}} = \exp(\gamma\,\mathrm{CC}(\mathrm{RF},\mathrm{IM})) \tag{11}$$

where $\mathrm{CC}(\mathrm{RF},\mathrm{IM})$ is the correlation coefficient of the image (IM) with the neuronal RF, and $\gamma = 3$ determines the sharpness of the exponential dependence. $\mathrm{CC}(\mathrm{RF},\mathrm{IM})$ was calculated for the central part of the image with the same size (in pixels) as RFs (that is, the central 200×200 pixels of the image for RFs instantiated on a visual field with 50×50 degrees extent and $\mathrm{ppd} = 4$).

The activity of the network was calculated after accounting for recurrent interactions, by applying the matrix operator $A = (I - W)^{-1}$ for different weight matrices: $r = [A\, I_{\mathrm{ffw}}]_+$. The gain of neuronal responses in response to each image is then obtained by dividing the activity of each neuron over its respective feedforward input. Discriminability of population responses to two images i and j are quantified by calculating the angle between the vectors of population responses (r_i and r_j):

$$\theta_{ij} = \frac{180}{\pi}\arccos\Big(\frac{< r_i.r_j >}{|r_i|.|r_j|}\Big) \tag{12}$$

where $< . >$ denotes the dot product and $|.|$ is the norm of the vector. $\theta_{ij} = 90$ corresponds to the maximum discriminability (orthogonal representations) and 0 or 180 degrees show the maximum collinear relationships (in the same or opposite directions, respectively). We use a

normalized version of this angle:

$$d_{ij} = |\theta_{ij} \bmod 90|/90 \tag{13}$$

to quantify discriminability (as in **Fig. 7H**), which ranges from 0 (minimum discriminability) to 1 (maximum discriminability).

To assess the decoding capacity of neuronal networks to discriminate natural images, we trained linear decoders on the population activity of the excitatory neurons. Each decoder was trained to distinguish a target image from other images. The ensemble of natural images was broken into two random parts (test and train sets, each containing 300 images) and the decoder was trained on the train set to detect the target image from the rest of the images. The training was done by presenting 300 pairs of images, containing the target image and one of the 300 test images. The decoder then finds (via linear regression) the best weighting of population activity of the network which separates the response to the target image from the non-target ones. To control for different levels of population activity under different conditions (e.g. networks with strong and weak recurrent interaction), we normalized the activity of the networks, such that the average activity of the network in response to each image was 1.

The decoder was tested on the test set, by presenting pairs of images containing the target image and each of the 300 test images. A threshold of 90% was set for the correct detection. The percent correct was then calculated as the fraction of the pairs for which the target image passed the threshold *and* the test image did not. The decoding task was performed for all 600 images as decoding targets. For each decoding task, the procedure was repeated for different levels of noise added to the population activity (both during training and for the test). It was added to the normalized activity of all excitatory neurons in response to each image and was drawn from a uniform distribution between 0 and ξ, with ξ ranging from 0.02 to 0.1 (**Fig. 7K**). The example shown in **Fig. 7J** had an intermediate noise level of 0.04.

2 Theoretical analysis of neuronal influence in single-neuron perturbations

We analytically evaluate the effect of single-neuron perturbations in networks of rate-based neurons as described above (Eq. (5)). We drop the firing threshold nonlinearity and analyze the linear behaviour of the network as described with the following dynamics:

$$\tau \frac{dr}{dt} = -r + Wr + s \tag{14}$$

The stationary state solution of the firing rates under such dynamics is obtained under $dr/dt = 0$ and can be written as:

$$r = Wr + s \rightarrow r = (I - W)^{-1}s. \tag{15}$$

We define $A = (I - W)^{-1}$ as the operator which acts on external input to obtain the steady-state firing rates in any equilibrium, $r_0 = As_0$. Single-cell perturbations around this steady-state leads to a new firing rate solution, $r_p = As_p$. Here, $s_p = s_0 + \delta s$, and δs is a vector of zeros at all entries except for the neuron which is perturbed. If the i-th neuron in the network is perturbed, we have:

$$\delta s_k = \begin{cases} 0, & k \neq i \\ \delta p, & k = i \end{cases} \tag{16}$$

where δp is the size of perturbation. To obtain the influence of perturbation of neuron i on the postsynaptic neuron j, $\psi(i \rightarrow j)$ we need to calculate the change in the firing rate of the j-th entry of r_p. Writing

$$\delta r = A \delta s \tag{17}$$

the rate change of the j-th neuron is obtained as:

$$\delta r_j = \sum_{k=1}^{N} A_{jk} \delta s_k = A_{ji} \delta s_i \tag{18}$$

where A_{ji} is the entry on the j-th row and i-th column of matrix A. Writing the influence as the rate change of the influencee j divided by the perturbation strength of the influencer i:

$$\psi(i \rightarrow j) = \frac{\delta r_j}{\delta s_i} = A_{ji} \tag{19}$$

reveals that A_{ji} is, in fact, denoting the influence.

To obtain the neuronal influence in single-neuron perturbations, we used the above framework to evaluate $\psi(i \rightarrow j) = A_{ji}$, by mathematically calculating the influence in networks with different profiles of connectivity. We explain this approach in more detail below.

2.1 Calculating influence for a general weight matrix

To obtain the influence, we calculate A_{ji}, by expanding the matrix A with regard to W as:

$$A = (I - W)^{-1} = I + W + W^2 + \ldots \tag{20}$$

A_{ji} can therefore be expressed as:

$$A_{ji} = \underbrace{W_{ji}}_{\psi^1(i \to j)} + \underbrace{(W^2)_{ji}}_{\psi^2(i \to j)} + \underbrace{(W^3)_{ji}}_{\psi^3(i \to j)} + \dots \quad (21)$$

Note that the first term in Eq. (20) (the identity matrix) does not contribute to the influence in Eq. (21), since $i \neq j$ (the influencer and the influencee are different neurons).

The series describes different pathways of interaction from i to j in the following fashion:

(I) Mono-synaptic influence denotes the direct interaction from i to j, which is inferred from the corresponding entry on the original weight matrix:

$$\psi^1(i \to j) = W_{ji} \quad (22)$$

(II) Di-synaptic influence entails second-order interactions, comprising all the pathways in which neuron i can influence neuron j via secondary neurons. It can be mathematically expressed as:

$$\psi^2(i \to j) = (W^2)_{ji} = \sum_{k=1}^{N} W_{jk} W_{ki} \quad (23)$$

where index k denotes all the neurons in the network that mediate the influence from neuron i to neuron j.

(III) Tri-synaptic influence captures all interactions with two layers of intermediate neurons, denoted by indices k and l in the following formulation:

$$\psi^3(i \to j) = (W^3)_{ji} = \sum_{l=1}^{N} W_{jl} (\sum_{k=1}^{N} W_{lk} W_{ki}) \quad (24)$$

Higher order interactions (including tetra-synaptic $\psi^4(i \to j)$, penta-synaptic $\psi^5(i \to j)$, etc) can be calculated via similar equations.

2.1.1 Networks with excitation and inhibition

In the next step, we calculated the influence for networks containing two subtypes of excitatory (E) and inhibitory (I) neurons, with the number of neurons in the network denoted by N_E and N_I, respectively. The connection weights from E to E, E to I, I to E and I to I neurons are described, respectively, by J_{EE}, J_{EI}, J_{IE}, and J_{II}. W is ordered such that the first N_E elements are excitatory neurons and the next N_I elements ($N_E + 1$ to $N_E + N_I$ rows/columns) represent inhibitory neurons.

The influence of the i-th excitatory neuron on the j-th excitatory neuron in the network can

be calculated as:

$$\psi(i \to j) = \psi^1(i \to j) + \psi^2(i \to j) + \psi^3(i \to j) + \dots \tag{25}$$

where different orders of influence are calculated as the following:

(I) Mono-synaptic influence is given by

$$\psi^1(i \to j) = W_{ji} = J_{EE} \tag{26}$$

which is the direct connection between the two excitatory neurons.

(II) Di-synaptic influence is calculated as

$$\begin{aligned} \psi^2(i \to j) =& \sum_{k=1}^{N_E} W_{jk}W_{ki} + \sum_{k'=N_E+1}^{N_E+N_I} W_{jk'}W_{k'i} \\ =& N_E J_{EE}^2 + N_I J_{EI} J_{IE} \end{aligned} \tag{27}$$

which contains pathways with either excitatory or inhibitory neurons in between the influencer and the influencee.

(III) Tri-synaptic influence can, in turn, be written as

$$\begin{aligned} \psi^3(i \to j) =& \sum_{l=1}^{N_E} W_{jl}(\sum_{k=1}^{N_E} W_{lk}W_{ki} + \sum_{k'=N_E+1}^{N_E+N_I} W_{lk'}W_{k'i}) \\ &+ \sum_{l'=N_E+1}^{N_E+N_I} W_{jl'}(\sum_{k=1}^{N_E} W_{l'k}W_{ki} + \sum_{k'=N_E+1}^{N_E+N_I} W_{l'k'}W_{k'i}) \end{aligned} \tag{28}$$

This includes four possibility of mediation between two excitatory neurons, $E \to X \to Y \to E$ (namely $E \to E \to E \to E$, $E \to E \to I \to E$, $E \to I \to E \to E$, $E \to I \to I \to E$, respectively) and can be calculated as:

$$\begin{aligned} \psi^3(i \to j) =& J_{EE}J_{EE}J_{EE}N_EN_E && (E \to E \to E \to E) \\ &+ J_{EE}J_{EI}J_{IE}N_EN_I && (E \to E \to I \to E) \\ &+ J_{EI}J_{IE}J_{EE}N_EN_I && (E \to I \to E \to E) \\ &+ J_{EI}J_{II}J_{IE}N_IN_I && (E \to I \to I \to E) \\ =& N_E^2 J_{EE}^3 + 2N_EN_IJ_{EI}J_{IE}J_{EE} + N_I^2 J_{EI}J_{IE}J_{II} \end{aligned} \tag{29}$$

Note that the latter motif entails a net positive influence, as it involves inhibition of inhibition.

(IV) Tetra-synaptic influence can, similarly, be mediated by 3-order motifs ($E \to X \to$

$Y \to Z \to E$, where $\{X, Y, Z\}$ can be either E or I, leading to a total of 8 possibilities), and therefore can be written as

$$
\begin{aligned}
\psi^4(i \to j) =& J_{EE}J_{EE}J_{EE}J_{EE} \times N_E N_E N_E && (E \to E \to E \to E \to E) \\
&+ J_{EE}J_{EE}J_{EI}J_{IE} \times N_E N_E N_I && (E \to E \to E \to I \to E) \\
&+ J_{EE}J_{EI}J_{IE}J_{EE} \times N_E N_I N_E && (E \to E \to I \to E \to E) \\
&+ J_{EE}J_{EI}J_{II}J_{IE} \times N_E N_I N_I && (E \to E \to I \to I \to E) \\
&+ J_{EI}J_{IE}J_{EE}J_{EE} \times N_I N_E N_E && (E \to I \to E \to E \to E) \\
&+ J_{EI}J_{IE}J_{EI}J_{IE} \times N_I N_E N_I && (E \to I \to E \to I \to E) \\
&+ J_{EI}J_{II}J_{IE}J_{EE} \times N_I N_I N_E && (E \to I \to I \to E \to E) \\
&+ J_{EI}J_{II}J_{II}J_{IE} \times N_I N_I N_I && (E \to I \to I \to I \to E)
\end{aligned}
\tag{30}
$$

Higher order influences can be calculated in a similar fashion by counting higher-order motifs.

2.1.2 The case of inhibition-dominance

It is useful to calculate the influence for a simplified description of the abovementioned weight matrices, where $J_{EE} = J$, $J_{EI} = \alpha J_{EE}$, and $J_{IE} = J_{II} = -gJ_{EE}$. We further assume $N_E = N_I = N$ and write:

$$
\begin{aligned}
\psi^1(i \to j) &= J \\
\psi^2(i \to j) &= NJ^2 - N\alpha JgJ = NJ^2(1 - \alpha g) \\
\psi^3(i \to j) &= N^2J^3 - 2N^2\alpha JgJJ + N^2\alpha JgJgJ = N^2J^3(1 - 2\alpha g + \alpha g^2) \\
\psi^4(i \to j) &= N^3J^4(1 - \alpha g - \alpha g + \alpha g^2 - \alpha g + \alpha^2 g^2 + \alpha g^2 - \alpha g^3) \\
&= N^3J^4(1 - 3\alpha g + 2\alpha g^2 + \alpha^2 g^2 - \alpha g^3)
\end{aligned}
\tag{31}
$$

For the specific condition that $\alpha = 1$, we have:

$$
\begin{aligned}
\psi^1(i \to j) &= J \\
\psi^2(i \to j) &= NJ^2(1 - g) \\
\psi^3(i \to j) &= N^2J^3(1 - g)^2 \\
\psi^4(i \to j) &= N^3J^4(1 - g)^3
\end{aligned}
\tag{32}
$$

which in fact provides a closed-form description of the influence at all orders of influence:

$$\psi^k(i \to j) = N^{k-1} J^k (1-g)^{k-1} \tag{33}$$

The total influence can therefore be written as:

$$\psi(i \to j) = \sum_k \psi^k(i \to j) = J(1 + NJ(1-g) + (NJ(1-g))^2 + \dots) \tag{34}$$

which can be expressed as:

$$\psi(i \to j) = \frac{J}{1 - NJ(1-g)} \tag{35}$$

Note that inhibition dominance, $g > 1$, does *not* imply a negative influence here. Rewriting $\kappa = 1 - NJ(1-g)$, and noticing that $\kappa > 1$ for $g > 1$, we observe *divisive inhibition* as a result of inhibition dominance:

$$\psi(i \to j) = \frac{J}{\kappa}. \tag{36}$$

The stronger the inhibition-dominance, the larger the divisive term in the denominator, and hence the higher the divisive inhibition.

2.2 Calculating influence for networks with specific connectivity

The calculations presented in the previous section can be extended to networks with specific connectivity, where the weight of connections between neurons are defined as a function of their functional similarity. First we consider a scenario where the functional property of neurons is defined by a one-dimensional parameter, e.g. their preferred orientations, θ. We consider weight matrices described by:

$$W_{ij} = J(1 + m \cos(2(\theta_i - \theta_j))) \tag{37}$$

where the connection weight between neuron i and j is modulated by the similarity of their respective preferred orientations. m determines the degree of specificity of connections, with $m = 0$ retrieving the unspecific weight matrices described in the previous section. We now calculate the influence (A_{ji}) for a network of excitatory and inhibitory neurons with specific

connectivity, described as:

$$\begin{aligned} W_{ij}^{EE} &= J_{EE}(1 + m_{EE}\cos(2(\theta_i^E - \theta_j^E))) \\ W_{ij}^{EI} &= J_{EI}(1 + m_{EI}\cos(2(\theta_i^E - \theta_j^I))) \\ W_{ij}^{IE} &= J_{IE}(1 + m_{IE}\cos(2(\theta_i^I - \theta_j^E))) \\ W_{ij}^{II} &= J_{II}(1 + m_{II}\cos(2(\theta_i^I - \theta_j^I))) \end{aligned} \tag{38}$$

Here, J_{XY} and m_{XY} denote, respectively, the average weight and the degree of specificity of synapses, and θ^X represents the preferred orientation. $(X, Y) \in (E, I)$.

We first calculate the influence for a scenario where all connections have the same degree of specificity, i.e. $m_{EE} = m_{EI} = m_{IE} = m_{II} = m$. We also assume that $J_{EE} = J$, $J_{EI} = \alpha J_{EE}$, and $J_{IE} = J_{II} = -gJ_{EE}$, as described above. Under these conditions, the influence of perturbing excitatory neuron i on the excitatory neuron j can be calculated as the following for different orders of interaction:

(I) Monosynaptic:

$$\psi^1(i \to j) = J(1 + m\cos(2\Delta\theta)) \tag{39}$$

where $\Delta\theta = \theta_i - \theta_j$.

(II) Di-synaptic:

$$\begin{aligned} \psi^2(i \to j) =& \sum_{k=1}^{N_E} W_{jk}W_{ki} + \sum_{k'=N_E+1}^{N_E+N_I} W_{jk'}W_{k'i} \\ =& \sum_{k=1}^{N_E} J(1 + m\cos(2(\theta_j - \theta_k))) \times J(1 + m\cos(2(\theta_k - \theta_i))) \\ &+ \sum_{k'=N_E+}^{N_E+N_I} (-gJ)(1 + m\cos(2(\theta_j - \theta_{k'}))) \times \alpha J(1 + m\cos(2(\theta_{k'} - \theta_i))) \end{aligned} \tag{40}$$

Assuming large N, we can solve the following continuous version of the equation:

$$\begin{aligned} \psi^2(i \to j) = & \int_{\theta=0}^{\pi} J^2(1 + m\cos(2(\theta - \theta_j)))(1 + m\cos(2(\theta - \theta_i)))d\theta \\ & - \int_{\theta=0}^{\pi} \alpha g J^2(1 + m\cos(2(\theta - \theta_j)))(1 + m\cos(2(\theta - \theta_i)))d\theta \end{aligned} \tag{41}$$

which, given $\Delta\theta = \theta_i - \theta_j$, leads to

$$\psi^2(i \to j) = NJ^2(1 - \alpha g)(1 + \frac{m}{2}\cos(2\Delta\theta)) \tag{42}$$

In calculating the above identity, we used the following equation:

$$\int_{\theta=0}^{\pi} (1 + m\cos(2(\theta - \theta_j)))(1 + m\cos(2(\theta - \theta_i)))d\theta = \pi(1 + \frac{m}{2}\cos(2(\theta_i - \theta_j))) \quad (43)$$

and its discrete equivalent:

$$\sum_{k=1}^{N}(1 + m\cos(2(\theta_k - \theta_j)))(1 + m\cos(2(\theta_k - \theta_i))) = N(1 + \frac{m}{2}\cos(2(\theta_i - \theta_j))) \quad (44)$$

It describes how indirect weights and their specificity are effectively determined when a layer of intermediate neurons is mediating the influence, and is useful to highlight since it appears recurrently in calculating higher-order motifs in what follows.

(III) Tri-synaptic:

$$\begin{aligned}\psi^3(i \to j) = &\sum_{l=1}^{N_E} W_{jl}(\sum_{k=1}^{N_E} W_{lk}W_{ki} + \sum_{k'=N_E+1}^{N_E+N_I} W_{lk'}W_{k'i}) \\ &+ \sum_{l'=N_E+1}^{N_E+N_I} W_{jl'}(\sum_{k=1}^{N_E} W_{l'k}W_{ki} + \sum_{k'=N_E+1}^{N_E+N_I} W_{l'k'}W_{k'i})\end{aligned} \quad (45)$$

which can be written as:

$$\begin{aligned}&\psi^3(i \to j) = \\ &\sum_{l=1}^{N_E} J(1 + m\cos(2(\theta_j - \theta_l))) \times \sum_{k=1}^{N_E}[J(1 + m\cos(2(\theta_j - \theta_k)))J(1 + m\cos(2(\theta_k - \theta_i)))]+ \\ &\sum_{l=1}^{N_E}(-gJ)(1 + m\cos(2(\theta_j - \theta_l))) \times \sum_{k'=N_E+1}^{N_E+N_I}[\alpha J(1 + m\cos(2(\theta_j - \theta_{k'})))J(1 + m\cos(2(\theta_{k'} - \theta_i)))]+ \\ &\sum_{l'=N_E+1}^{N_E+N_I} J(1 + m\cos(2(\theta_j - \theta_{l'}))) \times \sum_{k=1}^{N_E}[(-gJ)(1 + m\cos(2(\theta_j - \theta_k)))\alpha J(1 + m\cos(2(\theta_k - \theta_i)))]+ \\ &\sum_{l'=N_E+1}^{N_E+N_I}(-gJ)(1 + m\cos(2(\theta_j - \theta_{l'}))) \times \sum_{k'=N_E+1}^{N_E+N_I}[(-gJ)(1 + m\cos(2(\theta_j - \theta_{k'})))\alpha J(1 + m\cos(2(\theta_{k'} - \theta_i)))]\end{aligned} \quad (46)$$

and results in:

$$\psi^3(i \to j) = N^2J^3(1 - 2\alpha g + \alpha g^2)(1 + \frac{m}{4}\cos(2\Delta\theta)) \quad (47)$$

(IV) Tetra-synaptic: Accounting for all fourth order motifs in a similar fashion as explained

above, we obtain the following for the fourth order influence:

$$\psi^4(i \to j) = N^3 J^4 (1 - 3\alpha g + 2\alpha g^2 + \alpha^2 g^2 - \alpha g^3)(1 + \frac{m}{8}\cos(2\Delta\theta)) \tag{48}$$

The total influence between excitatory neurons i and j in specific EI networks can, therefore, be expressed as:

$$\begin{aligned}\psi(i \to j) =& (1 + m\cos(2\Delta\theta))J + \\ & (1 + \frac{m}{2}\cos(2\Delta\theta))NJ^2(1 - \alpha g) + \\ & (1 + \frac{m}{4}\cos(2\Delta\theta))N^2 J^3 (1 - 2\alpha g + \alpha g^2) + \\ & (1 + \frac{m}{8}\cos(2\Delta\theta))N^3 J^4 (1 - 3\alpha g + 2\alpha g^2 + \alpha^2 g^2 - \alpha g^3) + \\ & \dots\end{aligned} \tag{49}$$

Note that the influence contains nonspecific and specific components, and that the nonspecific component is similar to what we obtained before for influence in nonspecific networks (Eq. (31).

For the simplified case of $\alpha = 1$, we can follow similar steps as described above for nonspecific networks to characterize the influence for all higher orders with a closed form expression:

$$\begin{aligned}\psi(i \to j) =& (1 + m\cos(2\Delta\theta))J + \\ & (1 + \frac{m}{2}\cos(2\Delta\theta))NJ^2(1 - g) + \\ & (1 + \frac{m}{4}\cos(2\Delta\theta))N^2 J^3 (1 - 2g + g^2) + \\ & (1 + \frac{m}{8}\cos(2\Delta\theta))N^3 J^4 (1 - 3g + 2g^2 + \alpha^2 g^2 - g^3) + \\ & \dots \\ =& \sum_{k=1}^{N} (1 + \frac{m}{2^{k-1}}\cos(2\Delta\theta))N^{k-1} J^k (1 - g)^{k-1})\end{aligned} \tag{50}$$

The nonspecific part retrieves the same formulation as before (c.f. Eq. (35)):

$$\begin{aligned}\overline{\psi}(i \to j) &= J \sum_{k=0}^{\infty} N^k J^k (1 - g)^k \\ &= \frac{J}{1 - NJ(1 - g)}\end{aligned} \tag{51}$$

while the specific component of the influence can be expressed as:

$$\begin{aligned}\overline{\overline{\psi}}(i \to j) &= Jm\cos(2\Delta\theta) \sum_{k=0}^{\infty} N^k J^k (1-g)^k 2^{-k} \\ &= \frac{Jm\cos(2\Delta\theta)}{1 - NJ(1-g)/2}\end{aligned} \tag{52}$$

Note that inhibition dominance ($g > 1$) does *not* yield a net negative influence here too. That is, *feature-specific suppression* does not result from general inhibition dominance in specific EI networks, when $\alpha = 1$. Instead, it leads to (feature-specific) divisive inhibition (c.f. Eq. (35)), with higher inhibition-dominance values (g) implying higher divisive terms for the specific component.

2.2.1 Networks with broad inhibitory connectivity

So far, we considered the scenario where all connections had the same degrees of specific connectivity, by assuming $m_{EE} = m_{EI} = m_{IE} = m_{II} = m$. We now relax that assumption by allowing excitatory and inhibitory weights to have different degrees of specificity. We assume $m_{EE} = m_{EI} = m_e$ and $m_{IE} = m_{II} = m_i$, and solve for the condition where inhibition has a broader (i.e. less specific) connectivity, $m_e > m_i$.

Accounting for broader inhibition does not change the nonspecific component of the influence, but the specific component can now be written as:

$$\begin{aligned}\overline{\overline{\psi}}(i \to j) = &Jm_e\cos(2\Delta\theta) \\ &+ NJ^2(m_e^2 - gm_em_i)\cos(2\Delta\theta)/2 \\ &+ N^2J^3(m_e^3 - 2m_e^2gm_i + m_eg^2m_i^2)\cos(2\Delta\theta)/4 \\ &+ N^3J^4(m_e^4 - 3gm_e^3m_i + 3g^2m_e^2m_i^2 - g^3m_em_i^3)\cos(2\Delta\theta)/8 \\ &+ \dots\end{aligned} \tag{53}$$

Defining $J' = m_eJ$ and $g' = gm_i/m_e$, we can write:

$$\begin{aligned}\overline{\overline{\psi}}(i \to j) = &J'\cos(2\Delta\theta) \\ &+ NJ'^2(1 - g')\cos(2\Delta\theta)/2 \\ &+ N^2J'^3(1 - 2g' + g'^2)\cos(2\Delta\theta)/4 \\ &+ N^3J'^4(1 - 3g' + 3g'^2 - g'^3)\cos(2\Delta\theta)/8 \\ &+ \dots\end{aligned} \tag{54}$$

and therefore the specific component of the influence can be calculated as

$$\overline{\overline{\psi}}(i \to j) = J' \cos(2\Delta\theta) \sum_{k=0}^{\infty} N^k J'^k (1-g')^k 2^{-k} \tag{55}$$

which leads to:

$$\begin{aligned}\overline{\overline{\psi}}(i \to j) &= \frac{J' \cos(2\Delta\theta)}{1 - NJ'(1-g')/2} \\ &= \frac{Jm_e \cos(2\Delta\theta)}{1 - NJ(m_e - gm_i)/2}\end{aligned} \tag{56}$$

To obtain feature-specific suppression in single-neuron influences (namely, more suppression for pairs with smaller $\Delta\theta$), we need to have $1 - NJ(m_e - gm_i)/2 < 0$. Broader inhibition does not confer such a negative influence in inhibition-dominance networks, if $g\,m_i > m_e$. The only situation under which such a negative influence appears is if $m_e > gm_i$ and $NJ(m_e - gm_i)/2 > 1$ at the same time, but note that the latter condition implies instability of the weight matrix along the specific eigenmode.

2.3 Calculating influence for specific E-I networks with strong E-to-I connectivity

In this section, we relax the previously made assumption of $J_{EE} = J_{EI}$, and allow the excitatory neurons to have different connection weights to their excitatory and inhibitory postsynaptic targets, formulated by: $J_{EE} = J$, $J_{EI} = \alpha J_{EE}$, $J_{IE} = J_{II} = -gJ_{EE}$. We assume similar connection specificity for all synapses: $m_{EE} = m_{EI} = m_{IE} = m_{II} = m$. Following similar procedures as described before for networks with specific connectivity (and summarized in Eq. (49)), different orders of influence in specific networks with strong $E \to I$ connectivity can be written as:

$$\begin{aligned}
\psi^1(i \to j) &= J(1 + m\cos(2\Delta\theta)) \\
\psi^2(i \to j) &= NJ^2(1 - \alpha g)(1 + \frac{m}{2}\cos(2\Delta\theta)) \\
\psi^3(i \to j) &= N^2J^3(1 - 2\alpha g + \alpha g^2)(1 + \frac{m}{4}\cos(2\Delta\theta)) \\
\psi^4(i \to j) &= N^3J^4(1 - 3\alpha g + 2\alpha g^2 + \alpha^2 g^2 - \alpha g^3)(1 + \frac{m}{8}\cos(2\Delta\theta)) \\
&\vdots
\end{aligned} \tag{57}$$

We define $\psi_E = \psi_{E \to X \to E}$ and $\psi_I = \psi_{I \to X \to E}$ as two second-order, di-synaptic motifs of influence, one starting from an excitatory and the other from an inhibitory neuron, respectively.

The excitatory second-order motif ψ_E is $\psi^2(i \rightarrow j)$, by definition, and can hence be written as before:

$$\psi_E = NJ^2(1 - \alpha g)(1 + \frac{m}{2}\cos(2\Delta\theta)) \tag{58}$$

The inhibitory second-order motif can, in turn, be calculated as:

$$\psi_I = -NJ^2 g(1 - g)(1 + \frac{m}{2}\cos(2\Delta\theta)) \tag{59}$$

Note that, although starting from an inhibitory neuron, this motif would be positive for $g > 1$ (the condition which we referred to as inhibition-dominance). That is, inhibition-dominance leads to a net *excitatory* effect of the second-order motif of inhibitory neurons; this is because the net positive effect of inhibition of inhibition ($I \rightarrow I \rightarrow E$) is larger than the net negative effect of inhibition of excitation ($I \rightarrow E \rightarrow E$).

We define

$$\begin{aligned} \zeta_E &= (1 - \alpha g) \\ \zeta_I &= -g(1 - g) \end{aligned} \tag{60}$$

as the main factors appearing in the second-order excitatory and inhibitory motifs. This allows us to write the specific component of the higher-order motifs (in Eq. (57)) in terms of the basic factors of the respective di-synaptic motifs:

$$\begin{aligned} \overline{\overline{\psi^2}} &= \overline{\overline{\psi_E}} = \zeta_E \times NJ^2\frac{m}{2}\cos(2\Delta\theta) \\ \overline{\overline{\psi^3}} &= (\zeta_E + \alpha\zeta_I) \times N^2J^3\frac{m}{4}\cos(2\Delta\theta) \\ \overline{\overline{\psi^4}} &= (\zeta_E^2 - \frac{\alpha}{g}\zeta_I^2) \times N^3J^4\frac{m}{8}\cos(2\Delta\theta) \\ &\vdots \end{aligned} \tag{61}$$

Feature-specific suppression implicates a higher suppressive influence between neuronal pairs with smaller $\Delta\theta$. We can now evaluate this for specific components of higher-order motifs of influence, in view of the interaction of the basic di-synaptic motifs.

[1] For the 2nd-order motif, this implies that the basic excitatory second-order motif is negative, hence:

$$\zeta_E < 0 \rightarrow \alpha g > 1 \tag{62}$$

Both $E \rightarrow I$ connections (parameterized by α) and $I \rightarrow \{E, I\}$ weights (parameterized by g, or inhibition-dominance) can be strong to satisfy this condition, which highlights the significance of the specific excitatory-inhibitory interaction.

[2] For networks with weak recurrent coupling ($NJ \ll 1$), higher-orders of influence would

be much smaller than the lower orders and hence can be ignored in the net influence. However, higher-order motifs cannot be ignored in networks with strong coupling ($NJ \gg 1$). In that case, the condition inferred from the 2nd-order motif, $\zeta_E < 0$, does not guarantee a negative specific 3rd-order motif, since we need: $(\zeta_E + \alpha\zeta_I) < 0$. However, as we argued above, inhibition-dominance ($g > 1$) implies a positive ζ_I. ζ_I is only negative if we have:

$$\zeta_I < 0 \rightarrow g < 1 \tag{63}$$

This means that α and g cannot be arbitrarily increased as we assumed for the 2nd-order motif in [1].

An alternative way to satisfy this condition might be obtained by broadening of inhibition. If we allowed for different selectivity of excitatory and inhibitory connectivity (as denoted by m_e and m_i in Section. 2.2.1), the condition in Eq. (63) would change to $g\, m_i/m_e < 1$. Now, this can be satisfied by changing g and/or m_i/m_e, with the small values of the latter ($m_i/m_e < 1$) implying broaderer specificity of inhibitory connections compared to excitation. Overall, it means a weak specific inhibitory connectivity, which can be satisfied by weaker or broader inhibition. As strong inhibition-dominance might be necessary to balance nonspecific excitation, broader inhibition might be a better strategy to satisfy the negative influence of this specific motif.

[3] So far, negative influence of motifs could be satisfied, if the the two basic 2nd-order excitatory and inhibitory specific motifs were negative. For the 4th-order specific motif to be negative, we need:

$$\zeta_E^2 - \frac{\alpha}{g}\zeta_I^2 < 0 \tag{64}$$

We therefore have:

$$\alpha/g > \zeta_E^2/\zeta_I^2 \tag{65}$$

One way to achieve this is to have balanced di-synaptic motifs: $\zeta_E \approx \zeta_I$. Under this condition, we have $\zeta_E^2/\zeta_I^2 \approx 1$, and we therefore need $\alpha/g > 1$, or $\alpha > g$. But this is already satisfied, since the two conditions $\alpha g > 1$ (from Eq. (62)) and $g < 1$ (from Eq. (63)), imply $\alpha > 1 > g$ and hence $\alpha > g$.

Taken together, several conclusions can be inferred from our little exercise here: first, it is not possible to obtain negative influence for specific motifs by simply increasing the inhibition-dominance g; too large values of g can lead to strong disinhibitory effects, which might counteract the direct inhibitory effects via higher-order interactions. Second, the strength of $E \rightarrow I$ connections are as important, if not more, to achieve negative influence. Finally, balancing excitatory and inhibitory motifs might be needed, to avoid the dominance of one over the other.

However, although illustrative, these results are not conclusive, for the following reasons.

First, we do not have a closed-form expression for higher-order motifs, which hinders a systematic evaluation of all such terms and the conditions for their negativity. Moreover, the actual condition for the net negative influence is not the negativity of each higher-order term, but the negativity of the net sum. We therefore need mathematical formulations which provide us with these two requirements. We attempt to provide such analyses in the following sections.

2.3.1 Networks with dominant E-to-I connections only

It is instructive to study a special case where $\alpha \gg 1$ and $g = 1$, to evaluate the effect of strong E-to-I connections in the absence of strong inhibition-dominance. Under this condition, the basic di-synaptic motifs (Eq. (60)) can be written as

$$\begin{aligned} \zeta_E &= 1 - \alpha \\ \zeta_I &= 0 \end{aligned} \tag{66}$$

Only the excitatory component of basic di-synaptic motifs counts for calculating higher-order motifs, and we can therefore express different motifs as the following:

$$\begin{aligned} \psi^1 &= J(1 + m\cos(2\Delta\theta)) \\ \psi^2 &= J^2 N(1-\alpha)(1 + \frac{m}{2}\cos(2\Delta\theta)) \\ \psi^3 &= J^3 N^2(1-\alpha)(1 + \frac{m}{4}\cos(2\Delta\theta)) \\ \psi^4 &= J^4 N^3(1-\alpha)^2(1 + \frac{m}{8}\cos(2\Delta\theta)) \\ \psi^5 &= J^5 N^4(1-\alpha)^2(1 + \frac{m}{16}\cos(2\Delta\theta)) \\ \psi^6 &= J^6 N^5(1-\alpha)^3(1 + \frac{m}{32}\cos(2\Delta\theta)) \\ &\vdots \end{aligned} \tag{67}$$

Writing in terms of the sum of subsequent motifs ($\psi_1 + \psi_2$, $\psi_3 + \psi_4$, $\psi_5 + \psi_6$, $\ldots$), we reach to the following closed-form expression of the total influence, for nonspecific and specific components, respectively:

$$\begin{aligned} \overline{\psi} &= J[(1-\alpha)J^2N^2]^k(1 + (1-\alpha)JN) \\ \overline{\overline{\psi}} &= J[\frac{1-\alpha}{2}J^2N^2]^k(1 + \frac{1-\alpha}{2}JN)m\cos(2\Delta\theta)) \end{aligned} \tag{68}$$

and hence:

$$\begin{aligned}\overline{\psi} &= \frac{J[1+(1-\alpha)JN]}{1-J^2N^2(1-\alpha)} \\ \overline{\overline{\psi}} &= \frac{J[1+\frac{1-\alpha}{2}JN]m\cos(2\Delta\theta)}{1-J^2N^2\frac{1-\alpha}{2}}\end{aligned} \tag{69}$$

If $\alpha > 1$, the denominator is positive, and the condition for a net negative influence becomes:

$$\begin{aligned}&1+(1-\alpha)JN < 0 \\ &\alpha > 1+\frac{1}{JN}\end{aligned} \tag{70}$$

for the nonspecific, and

$$\alpha > 1+\frac{2}{JN} \tag{71}$$

for the specific component of the influence. Both conditions can be met by strong E-to-I (α) and/or strong connectivity (JN), but α should be stronger for a net negative specific influence.

2.3.2 Solution for strong E-to-I and inhibitory connections

Here, we consider a more general conditions, where $E \to I$ and $I \to \{E, I\}$ connections are both arbitrary and strong (i.e. $J_{EE} = J$, $J_{EI} = \alpha J_{EE}$, $J_{IE} = J_{II} = -gJ_{EE}$, and $g > 0$, $\alpha > 0$). We start by writing the n-th excitatory and inhibitory motifs of influence (i.e., the motifs starting from an n-th order E or I neurons in the chain of influence), in terms of the subsequent motifs:

$$\begin{aligned}\psi_E^n &= NJ\psi_E^{n-1} + \alpha NJ\psi_I^{n-1} \\ \psi_I^n &= -NgJ\psi_E^{n-1} - NgJ\psi_I^{n-1}\end{aligned} \tag{72}$$

Replacing inhibitory motifs from the second equation iteratively, the n-th excitatory motif can be written in terms of lower order excitatory motifs as:

$$\psi_E^n = NJ\psi_E^{n-1} + \alpha(-g)(NJ)^2\psi_E^{n-2} + \alpha(-g)^2(NJ)^3\psi_E^{n-2} + \dots \tag{73}$$

which can be described by the following recursive formula:

$$\begin{aligned}&\psi_E^n = NJ\psi_E^{n-1} + \alpha\sum_{k=1}^{n-2}(-g)^{n-k-1}(NJ)^{n-k}\psi_E^k + \alpha(-gNJ)^{n-1}J \\ &\psi_E^1 = J\end{aligned} \tag{74}$$

If we write Eq. (72) in the matrix form:

$$\begin{pmatrix} \psi_E^n \\ \psi_I^n \end{pmatrix} = \underbrace{NJ \begin{pmatrix} 1 & \alpha \\ -g & -g \end{pmatrix}}_{\Phi} \begin{pmatrix} \psi_E^{n-1} \\ \psi_I^{n-1} \end{pmatrix} \tag{75}$$

the k-th motif can be obtained by applying the matrix Φ, recursively, on lower orders:

$$\begin{pmatrix} \psi_E^k \\ \psi_I^k \end{pmatrix} = \Phi^{k-1} \begin{pmatrix} \psi_E^1 \\ \psi_I^1 \end{pmatrix} = \Phi_{11}^{k-1} J - \Phi_{12}^{k-1} gJ \tag{76}$$

Here, Φ_{11}^k and Φ_{12}^k are the entries on the first row and the first and the second columns, respectively, of the k-th power of Φ, and we have used the identities $\psi_E^1 = J$ and $\psi_I^1 = -gJ$. The influence of an excitatory neuron i on a target neuron j can now be calculated by counting the influence via all such higher order motifs:

$$\psi_E(i \to j) = \sum_{k=0}^{\infty} (\Phi_{11}^k J - \Phi_{12}^k gJ) \tag{77}$$

Defining $A^\Phi = \sum_{k=0}^{\infty} \Phi^k = (I - \Phi)^{-1}$, we can write:

$$\psi_E(i \to j) = A_{11}^\Phi \, J - A_{12}^\Phi \, gJ \tag{78}$$

A^Φ can be calculated as:

$$A^\Phi = \frac{1}{(1 - NJ)(1 + gJN) + \alpha NJgNJ} \begin{pmatrix} 1 + gNJ & \alpha NJ \\ -gNJ & 1 - NJ \end{pmatrix} \tag{79}$$

and hence the influence can be written as:

$$\psi_E(i \to j) = \frac{J + gNJ^2(1 - \alpha)}{1 + (g - 1)NJ + g(\alpha - 1)(NJ)^2} \tag{80}$$

Strong g and α would make the denominator positive, but for a negative influence the numerator should be negative, which, assuming strong connectivity and hence $gNJ \gg 1$, necessitates the condition of $\alpha > 1$. Hence, strong $E \to I$ connections is necessary to obtain a negative influence. Similar arguments can be made for the tuned component of influence, which argues for the necessity of strong $E \to I$ specific connectivity in order to obtain feature-specific suppression in single-neuron perturbations.

2.4 Solution for general connectivity conditions

Here, we consider the most general condition, where all connections ($E \rightarrow \{E, I\}$ and $I \rightarrow \{E, I\}$) can have arbitrary weights. We parameterize this condition by defining: $J_{EE} = J$, $J_{EI} = \alpha J_{EE}$, $J_{IE} = -\beta g J_{EE}$, and $J_{II} = -g J_{EE}$. Dominance of weights with regards to the excitatory weight, J_{EE}, is thus determined by three independent factors: α denoting the dominance of $E \rightarrow I$; g denoting the dominance of inhibition; and β parameterizing the extra inhibition of $I \rightarrow E$ weights. All factors can indicate dominance (> 1) or lack thereof (< 1).

The n-th excitatory and inhibitory motif of influence can be expressed now in terms of previous motifs (similar to Eq. (72)), in a recursive fashion:

$$\begin{aligned} \psi_E^n &= NJ\psi_E^{n-1} + \alpha NJ\psi_I^{n-1} \\ \psi_I^n &= -\beta NgJ\psi_E^{n-1} - NgJ\psi_I^{n-1} \end{aligned} \tag{81}$$

Expressing in matrix form as before (Eq. (75)), we have:

$$\begin{pmatrix} \psi_E^n \\ \psi_I^n \end{pmatrix} = \underbrace{NJ \begin{pmatrix} 1 & \alpha \\ -\beta g & -g \end{pmatrix}}_{\Phi} \begin{pmatrix} \psi_E^{n-1} \\ \psi_I^{n-1} \end{pmatrix}. \tag{82}$$

The k-th order motif can now be obtained by recursive application of operator Φ on all lower-order ($< k$) motifs, resulting in:

$$\begin{pmatrix} \psi_E^k \\ \psi_I^k \end{pmatrix} = \Phi^{k-1} \begin{pmatrix} \psi_E^1 \\ \psi_I^1 \end{pmatrix} = \Phi_{11}^{k-1} J - \Phi_{12}^{k-1} \beta g J \tag{83}$$

The influence of an excitatory neuron i on a target neuron j can again be calculated by counting the influence via all higher order motifs:

$$\psi_E(i \rightarrow j) = \sum_{k=0}^{\infty} (\Phi_{11}^k J - \Phi_{12}^k \beta g J) \tag{84}$$

which can, in turn, be written as:

$$\psi_E(i \rightarrow j) = A_{11}^{\Phi} J - A_{12}^{\Phi} \beta g J \tag{85}$$

where

$$A^{\Phi} = \sum_{k=0}^{\infty} \Phi^k = (I - \Phi)^{-1}. \tag{86}$$

A^{Φ} can now be calculated as:

$$A^{\Phi} = \frac{1}{(1 - NJ)(1 + gJN) + \alpha\beta NJgNJ} \begin{pmatrix} 1 + gNJ & \alpha NJ \\ -\beta gNJ & 1 - NJ \end{pmatrix} \tag{87}$$

The influence can therefore be expressed as:

$$\psi_E(i \to j) = \frac{J + gNJ^2(1 - \alpha\beta)}{1 + (g - 1)NJ + g(\alpha\beta - 1)(NJ)^2} \tag{88}$$

Following the same argument made for Eq. (80), we reach to the conclusion that, given a strong level of inhibition-dominance ($g \gg 1$), the condition for negative influence is $\alpha\beta > 1$. This argues for a strong interaction of dominant $E \to I$ and $I \to E$ weights as the necessary condition for suppressive influence of single-neuron perturbations. Similar formulation and argument for the case of specific connectivity argues for strong and specific connectivity of $E \to I$ and $I \to E$ as a prerequisite for suppressive influence along the specific dimension, and hence *feature-specific suppression*. This, in turn, explains why global inhibition dominance, or broad inhibition, alone is not enough for feature-specific suppression in EI networks.
